# Access to 6-hydroxy indolizines and related imidazo[1,5-*a*]pyridines through the S_N_2 substitution/condensation/tautomerization cascade process†

**DOI:** 10.1039/d1ra04425g

**Published:** 2021-07-23

**Authors:** Guiyun Duan, Hao Liu, Liqing Zhang, Chunhao Yuan, Yongchao Li, Yanqing Ge

**Affiliations:** Department of Pharmacy, Shandong First Medical University, Shandong Academy of Medical Sciences Taian Shandong 271016 P. R. China geyanqing2016@126.com +86-538-6229741 +86-538-6229741

## Abstract

A simple and efficient cascade reaction was developed for the construction of hydroxy substituted indolizines from pyrrole-2-carbaldehydes and commercially available 4-halogenated acetoacetic esters. Their optical properties were also evaluated.

## Abstract 2

A simple and efficient cascade reaction was developed for the construction of hydroxy substituted indolizines from pyrrole-2-carbaldehydes and commercially available 4-halogenated acetoacetic esters.

## Graphical abstract

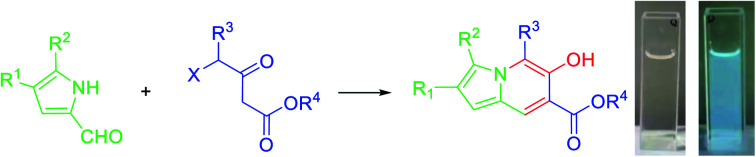

## Introduction

Indolizine, a biostere for indole, is commonly found in numerous natural products and pharmaceuticals. Indolizine derivatives exhibit diverse biological activities such as anti-HIV, anti-inflammatory, anti-tubercular, and anticancer activities.^1–5^ They are also used in dyes and optical materials owing to their bright colors.^6–11^ As a consequence, much effort has been devoted to their synthesis and functionalization, and thus many methods have been developed.^12–14^ In addition to classical Scholtz or Tschichibabin reactions, a variety of straightforward and efficient methods have been reported in recent years^15–20^ including 1,3-dipolar cycloaddition of pyridinium salts and intramolecular cyclization catalyzed by transition metals and intermolecular cyclization. Despite the efficiency of these methods, they suffer from the requirement of specific preorganized substrates, necessity of expensive metal catalysts, multistep synthesis, and a lack of product diversity. Moreover, no method has been reported for the preparation of indolizines bearing a hydroxyl group.

**Scheme 1:**
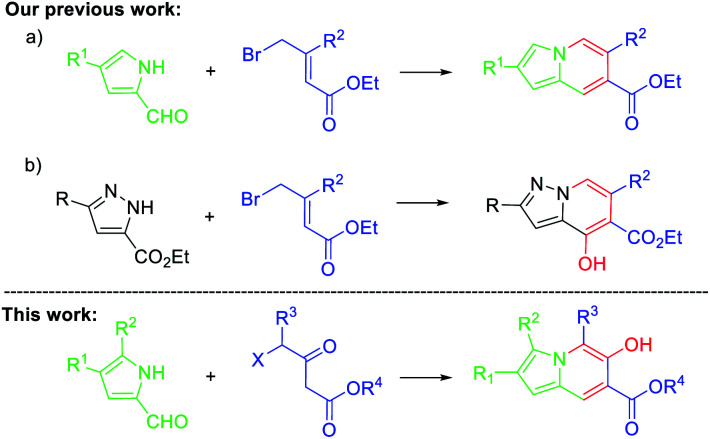
Access to indolizines *via* a cascade reaction.

Recently, we synthesized a series of indolizine and related N-bridgehead heterocycles *via* a cascade reaction (Scheme 1a).^21^ To achieve the related pyrazolo[1,5-*a*]pyridines through a shorter and convenient route, a simple and efficient synthetic method was also reported subsequently using commercially accessible starting materials (Scheme 1b).^22^ Based on the results obtained in our laboratory, we expected that a cascade reaction of pyrrole-2-carbaldehyde 1 with 4-halogenated acetoacetic ester 2 might be successful in the presence of a weak base (Scheme 1). In continuation of our effort to search for new fluorophores for imaging,^23–26^ herein, we report a simple and efficient method for the synthesis of hydroxy substituted indolizines *via* an S_N_2 substitution/condensation/tautomerization cascade process in a metal-free fashion. Their optical properties were also evaluated.

## Results and discussion

Initially, commercially available pyrrole-2-formaldehyde and ethyl 4-chloro-3-oxobutanoate 2a were selected for the experimental design. However, only the dimerization product of 2a was obtained. Subsequently, we optimized the reaction conditions using 4-propionyl-pyrrole-2-formaldehyde 1a and ethyl 4-chloro-3-oxobutanoate 2a as the model substrates. To our delight, the desired cyclized product 3a was obtained in an acceptable 56% yield in the presence of K_2_CO_3_ (Table 1, entry 1). The reaction even progressed very well at room temperature (entry 2). Other bases such as DBU, Cs_2_CO_3_, NaOH, *t*-BuOK, NaOAc, and CsOAc were then evaluated. However, the yields decreased, especially for a weak base (NaOAc) (entries 3–8). Regarding the effect of solvents, no improvement in the yield was obtained when the reaction was carried out in DMF, EtOH, and acetone (entries 9–11).

**Table 1:** Optimization of reaction conditions^a^

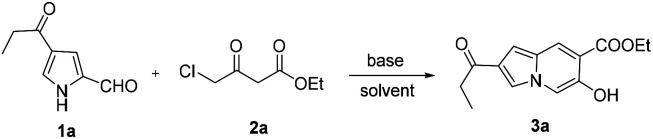					
Entry	Base	*T* (°C)	Solvent	Time	Yield (%)
1	K_2_CO_3_	50	MeCN	6 h	56
**2**	**K**_**2**_ **CO**_**3**_	**25**	**MeCN**	**6 h**	**70**
3	DBU	25	MeCN	6 h	54
4	Cs_2_CO_3_	25	MeCN	6 h	63
5	NaOH	25	MeCN	6 h	42
6	*t*-BuOK	25	MeCN	6 h	59
7	NaOAc	25	MeCN	6 h	12
8	CsOAc	25	MeCN	6 h	26
9	K_2_CO_3_	25	DMF	6 h	67
10	K_2_CO_3_	25	EtOH	6 h	60
11	K_2_CO_3_	25	Acetone	6 h	62

a 1 mmol 1a, 2 mmol 2a, 3 mmol base, and 10 mL solvent were used.

**Scheme 2:**
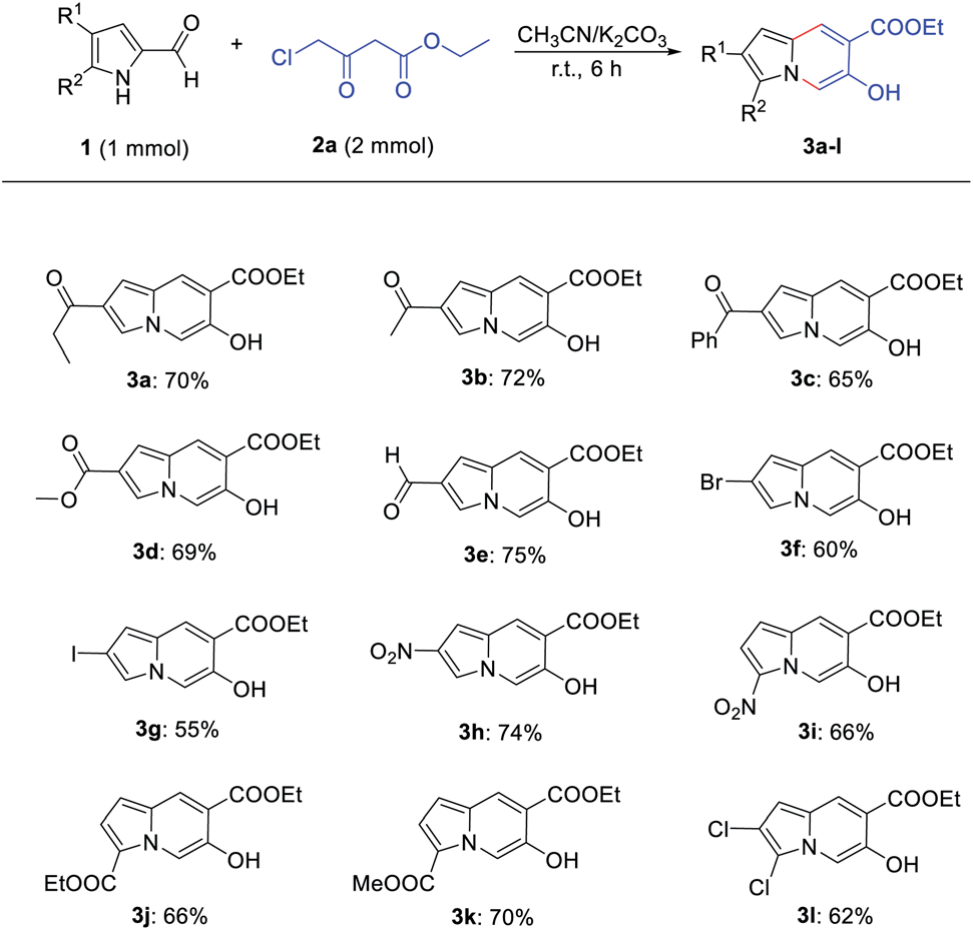
Substrate scope of pyrrole-2-formaldehyde. Reaction conditions: 1 (1 mmol), 2 (2 mmol), K_2_CO_3_ (3 mmol), CH_3_CN (10 mL), 20 °C, 6 h.

**Fig. 1:**
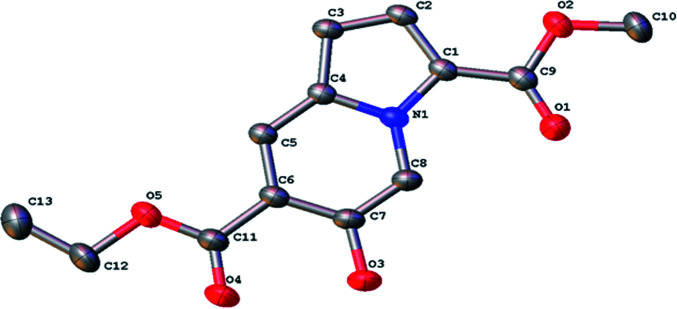
X-ray crystal structure of compound 3k.

Then, the reactions of various substituted pyrrole-2-formaldehyde were tested (Scheme 2). Generally, the desired products were obtained in moderate-to-good yields when the pyrroles contained electron-withdrawing groups at the 4- or 5-position. However, no product was obtained for pyrrole-2-formaldehyde, presumably due to the reduction of nucleophilicity of the pyrrole ring. The structure of compound 3k was confirmed by X-ray crystal structure analysis (Fig. 1, CCDC 2081693†).

**Scheme 3:**
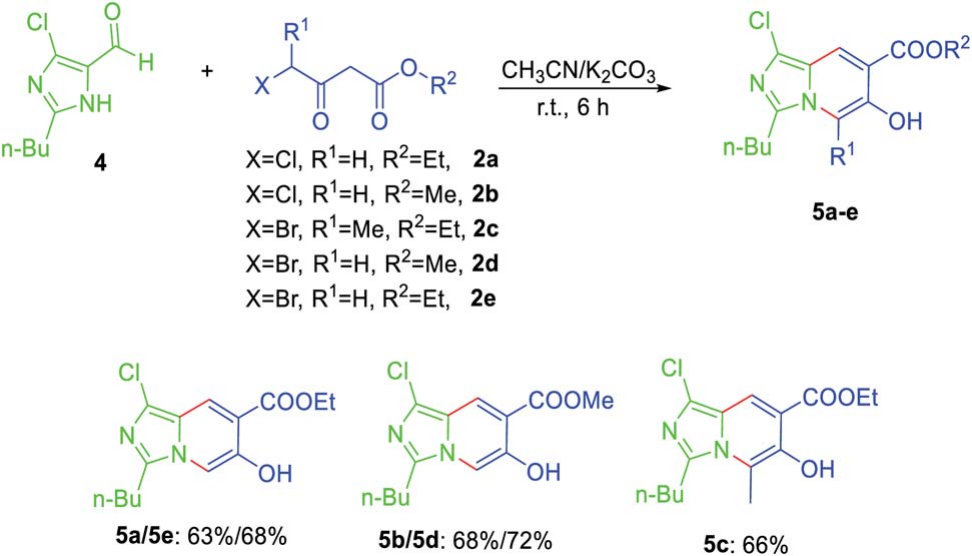
Substrate scope studies of 4-halogenated acetoacetic ester. Reaction conditions: 4 (1 mmol), 2 (2 mmol), K_2_CO_3_ (3 mmol), CH_3_CN (10 mL), 20 °C, 6 h.

Next, the applicability of this cascade reaction was expanded to the synthesis of imidazo[1,5-*a*]pyridine, furnishing the desired product 5a in 61% yield. The scope of the 4-halogenated acetoacetic ester was also evaluated (Scheme 3). The results indicate that the halogen and alkyl groups on position 4 and the ether group hardly influenced the yields.

**Scheme 4:**
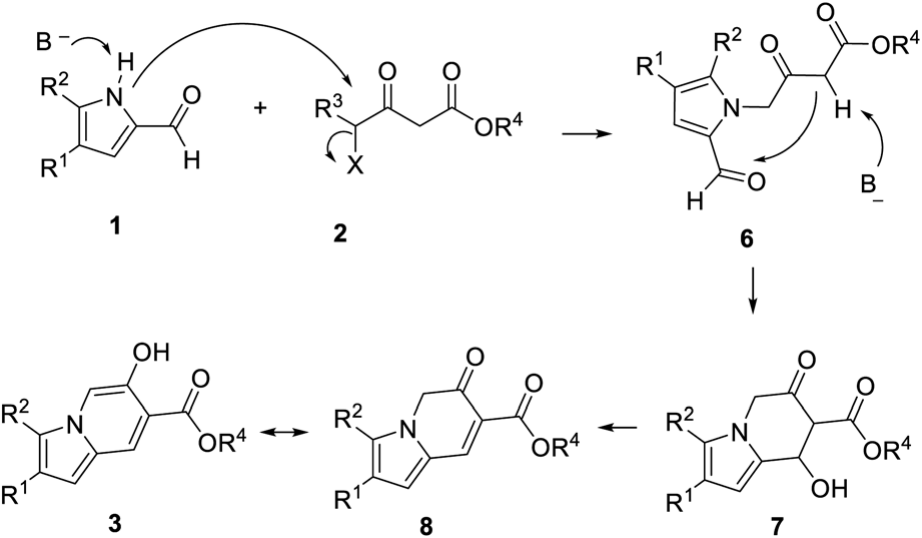
The proposed mechanism.

Based on the above mentioned results and our previous work, we propose a mechanism as shown in Scheme 4. First, S_N_2 substitution of 4-halogenated acetoacetic ester 2 and pyrrole-2-formaldehyde 1 yields intermediate 6. Subsequently, cyclized intermediate 7 is formed through intramolecular nucleophilic substitution. Finally, the desired products 3 are obtained through dehydration and tautomerism.

**Fig. 2:**
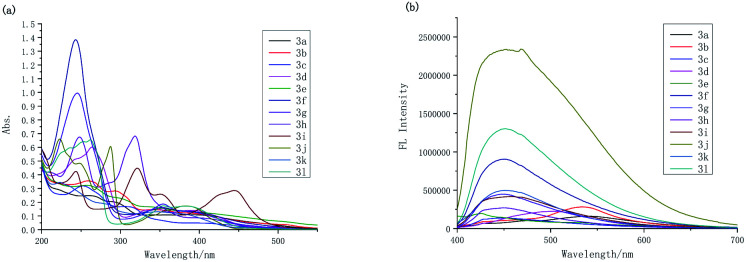
UV-vis and FL spectra of compounds 3a–3l.

To advance our efforts to search for new fluorophores for cell imaging and their strong luminescence, we investigated the UV-vis and fluorescence spectra of these new compounds (Fig. 2). Compounds 3a–3l show similar absorptions at *ca.* 250 nm (Table S1†), which should be assigned to the π–π* electronic transition originating from the indolizine ring. Notably, the substituent and their position on the indolizine ring slightly affect these absorption peaks. However, the weak absorption bands between 290 nm and 445 nm due to n–π* electronic transition are especially different for compounds 3h and 3i containing a strong electron-withdrawing group (NO_2_). The maximum emission bands of 3c, 3e, and 3f are similar (425–455 nm, Table S1†), while those of 3a, 3b, and 3d are 540 nm, 535 nm, and 505 nm, respectively, with a much higher red shift.

## Conclusions

In summary, we developed an efficient cascade reaction to construct indolizines and related imidazo[1,5-*a*]pyridines with a hydroxyl group, which is difficult to introduce through other methods. The structure was confirmed by single-crystal X-ray diffraction analysis. The compounds showed strong fluorescence in a dilute solution. Further studies on the optical properties of these indolizines are in progress.

## Conflicts of interest

There are no conflicts to declare.

## Supplementary Material

RA-011-D1RA04425G-s001

RA-011-D1RA04425G-s002
