# Supplementary material for: Access to 6-hydroxy indolizines and related imidazo[1,5-a]pyridines through the SN2 substitution/condensation/tautomerization cascade process
Source: RSC Adv. 2021 Jul 23;11(41):25624–7. doi: 10.1039/d1ra04425g (PMC9036982; doi:10.1039/d1ra04425g)
Supplement: RA-011-D1RA04425G-s001 [file RA-011-D1RA04425G-s001.pdf]

Electronic Supplementary Material (ESI) for RSC Advances.
This journal is © The Royal Society of Chemistry 2021

# Access to 6-hydroxy indolizines and related imidazo[1,5-a]pyridines through the $S_N2$ substitution/condensation/tautomerization cascade process

Guiyun Duan[1], Hao Liu[1], Liqing Zhang, Chunhao Yuan, Yongchao Li, Yanqing Ge*

*Department of Chemistry and Pharmaceutical Engineering, Shandong First Medical University, Shandong Academy of Medical Sciences, Taian, Shandong 271016, P. R. China*

**Corresponding Author:** Yanqing Ge

E-mail address: geyanqing2016@126.com

Tel.: +86-538-6229741; Fax: +86-538-6229741

**Table of Contents:**

Experimental section **S3**

Reference **S14**

Table S1 The optical characteristics of the compounds **3a-l** in EtOH **S14**

$^{1}$H NMR spectrum of compound **1a-f**, **1h**, **1i**, **1k** and **1l** **S15**

$^{1}$H NMR, $^{13}$C NMR and HRMS spectrum of compound **3a-l** and **5a-c** **S19**

**Experimental section**

General

$^{1}H$ NMR and $^{13}C$ NMR spectra were measured on a Bruker Avance 400 (400 MHz) spectrometer ($CDCl_3$ or DMSO-$d_6$ as solvent and tetramethylsilane (TMS) as an internal standard). High-resolution mass spectra (HRMS) were measured on an Agilent 6546 LC/Q-TOF spectrometer using electron spray ionization (ESI) technique. Chromatographic separations were done by column chromatography using 200-300 mesh silica gel. UV-vis spectra and fluorescence spectra were recorded on a UV-2600 spectrometer (Shimadzu) and FS-5 luminescence spectrophotometer (Edinburg) at room temperature, respectively. All reagents and solvents were purchased from commercial sources and used without further purification. 4-Iodo-*1H*-pyrrole-2-carbaldehyde **1g** and ethyl 5-formyl-*1H*-pyrrole-2-carboxylate **1j** were obtained from Innochem.

*Preparation of 4-propionyl-1H-pyrrole-2-carbaldehyde **1a***

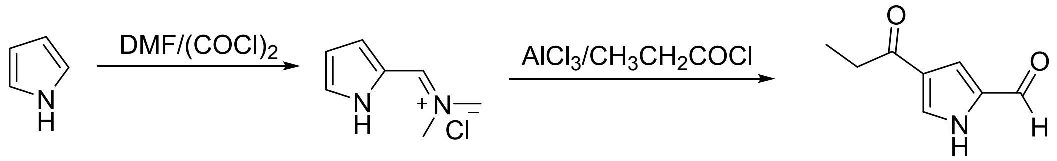


The literature procedure was followed with some modification.[1-2] A solution of dimethylformamide (2.01 g, 27.5 mmol) in 1, 2-dichloroethane (10 mL) in a 3-necked flask was cooled in an ice bath. To the stirred and cooled solution was added a solution of oxalyl chloride (3.49g, 27.5 mmol) in 1, 2-dichloroethane (4 mL) over a period of 20 min. The suspension of white solid was then allowed to stir at room temperature for 1 h. The suspension was cooled in ice and a solution of pyrrole (1.68 g, 25 mmol) in 1, 2-dichloroethane (5 mL) was added over 30 min. The light orange solution obtained was allowed to stir 1 h at room temperature. To this was added aluminum chloride (7.34 g, 55 mmol), followed by propionyl chloride (2.31 g, 25 mmol), rapidly and at room temperature. A small amount of warming occurred and the solution soon darkened. After stirred for 12 h, the mixture was poured onto 200 mL of ice and water, 50% aqueous NaOH solution (20 mL) was added, and the

mixture was stirred rapidly for 30 min. The mixture was then made slightly acidic (pH = 4) with concentrated hydrochloric acid. The organic and aqueous layers were separated. The aqueous layer was extracted with $CH_2Cl_2$ (60 mL × 10). The combined organic layer and $CH_2Cl_2$ extract was dried ($Na_2SO_4$), evaporated, and the crude product was purified by column chromatography (hexane/EA = 5/1) to afford **2a** (2.87 g, 76%) as a grey solid. $^1$H NMR (400 MHz, $CDCl_3$) δ 10.60 (s, 1H), 9.59 (s, 1H), 7.75 (t, $J$ = 1.6 Hz, 1H), 7.41 (t, $J$ = 1.6 Hz, 1H), 2.85 (q, $J$ = 7.2 Hz, 2H), 1.22 (t, $J$ = 7.2 Hz, 3H).

*Preparation of 4-acetyl-1H-pyrrole-2-carbaldehyde **1b**.*

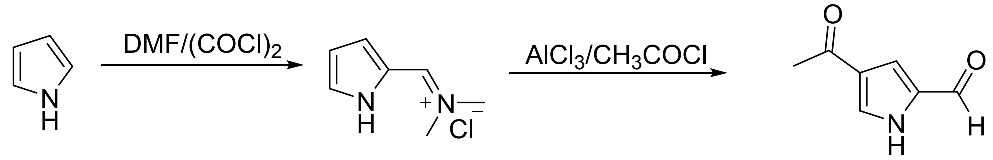


According to the synthesis of compound **2a**, the title compound was synthesized from pyrrole (1.68 g, 25 mmol) and acetyl chloride (1.96 g, 25 mmol). The product mixture was purified by silica gel column chromatography (hexane/EA = 5/1) to afford **2b** (2.47 g, 72%) as a yellow solid. $^1$H NMR (400 MHz, $CDCl_3$) δ 10.79 (s, 1H), 9.60 (s, 1H), 7.71 (d, $J$ = 1.6 Hz, 1H), 7.38 (s, 1H), 2.48 (s, 3H).

*Preparation of 4-benzoyl-1H-pyrrole-2-carbaldehyde **1c**.*

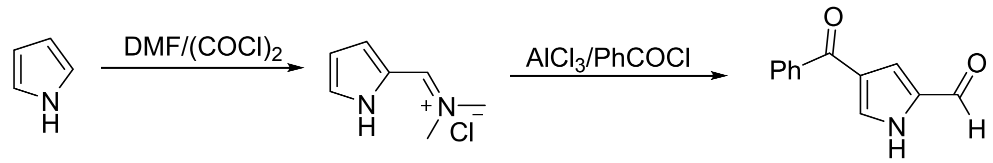


According to the synthesis of compound **2a**, the title compound was synthesized from pyrrole (1.68 g, 25 mmol) and benzoyl chloride (3.51 g, 25 mmol). The product mixture was purified by silica gel column chromatography (hexane/EA = 5/1) to afford **2c** (4.08 g, 82%) as a yellow solid.$^1$H NMR (400 MHz, $CDCl_3$) δ 10.55 (s, 1H), 9.62 (s, 1H), 7.85 (dd, $J$ = 8.0, 1.6 Hz, 2H), 7.73 (m, 1H), 7.59 (m, 1H), 7.50 (m, 3H).

*Preparation of methyl 5-formyl-1H-pyrrole-3-carboxylate* ***1d***.

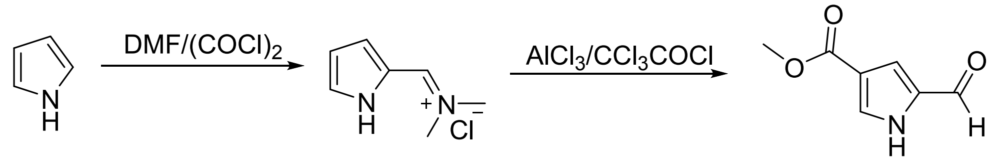


The literature procedure was followed with some modification.[1-2] A solution of dimethylformamide (2.01 g, 27.5 mmol) in 1, 2-dichloroethane (10 mL) in a 3-necked flask was cooled in an ice bath. To the stirred and cooled solution was added a solution of oxalyl chloride (3.49 g, 27.5 mmol) in 1, 2-dichloroethane (4 mL) over a period of 20 min. The suspension of white solid was then allowed to stir at room temperature for 1 h. The suspension was cooled in ice and a solution of pyrrole (1.68 g, 25 mmol) in 1, 2-dichloroethane (5 mL) was added over 30 min. The light orange solution obtained was allowed to stir 1 h at room temperature. To this was added aluminum chloride (7.34 g, 55 mmol), followed by trichloroacetyl chloride (4.55 g, 25 mmol), rapidly and at room temperature.

After stirred for 12 h at room temperature, methanol (50 mL) was added and, after 5 min stirring, solution of sodium (4.00 g, 0.175 mol) in methanol (50 mL was added and stirring continued for 2 h. The reaction product was then poured into cold water, stirred 10 min, and acidified with concentrated hydrochloric acid. The mixture was extracted with $CH_2Cl_2$ (60 mL × 10). The combined organic layer was dried ($Na_2SO_4$), evaporated, and the crude product was purified by column chromatography (hexane/EA = 5/1) to afford **2d** (2.87 g, 75%) as a white solid. $^1$H NMR (400 MHz, $CDCl_3$) δ 10.20 (s, 1H), 9.57 (s, 1H), 7.70 (d, $J$ = 1.6 Hz, 1H), 7.39 (s, 1H), 3.86 (s, 3H).

*Preparation of 1H-pyrrole-2,4-dicarbaldehyde* ***1e***.

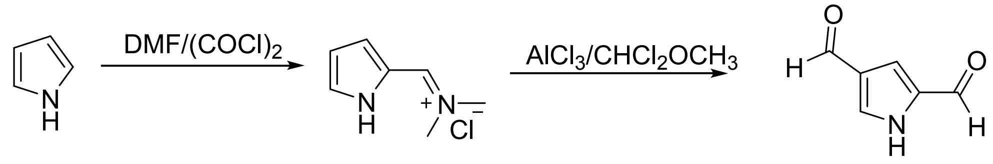


The literature procedure was followed with some modification.[1-2] A solution of dimethylformamide (2.01 g, 27.5 mmol) in 1, 2-dichloroethane (10 mL) in a 3-necked flask was cooled in an ice bath. To the stirred and cooled solution was added a

solution of oxalyl chloride (3.49 g, 27.5 mmol) in 1, 2-dichloroethane (4 mL) over a period of 20 min. The suspension of white solid was then allowed to stir at room temperature for 1 h. The suspension was cooled in ice and a solution of pyrrole (1.68 g, 25 mmol) in 1, 2-dichloroethane (5 mL) was added over 30 min. The light orange solution obtained was allowed to stir 1 h at room temperature. To this was added aluminum chloride (7.34 g, 55 mmol), followed by nitromethane (3.66 g, 60 mmol), rapidly and at room temperature. Then the mixture was cooled to 0 °C. Dichloromethyl methyl ether (2.88 g, 25 mmol) was added all at once and the cooling bath removed shortly after. The solution was stirred for 12 h at room temperature and worked up in the usual manner giving a yellow-brown solid. The crude product was purified by column chromatography (hexane/EA = 5/1) to afford **2e** (1.85 g, 60%) as a yellow solid. $^1$H NMR (400 MHz, $CDCl_3$) δ 10.18 (s, 1H), 9.89 (s, 1H), 9.64 (s, 1H), 7.72 (d, $J$ = 2.0 Hz, 1H), 7.42 (s, 1H).

*Preparation of 4-bromo-1H-pyrrole-2-carbaldehyde **1f**.*

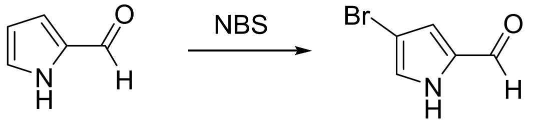


The literature procedure was followed with some modification.[3] A solution of pyrrole-2-carboxaldehyde (3.80 g, 40 mmol) in THF (40 m) was cooled to 0 °C under nitrogen in a 100 mL round bottom flask. NBS (7.12 g, 40 mmol) was added all at once. The reaction mixture was stirred for 15 min at 0 °C under nitrogen. The solvent was removed on a rotary evaporator. Water (20 mL) was added to the flask and the suspension was filtered. The filter cake was washed with an additional 20 mL of water. The solid was crystallized from the EtOH/$H_2O$ solution (100 mL, EtOH/$H_2O$ = 1:9) to give 4.82g of product (69%). $^1$H NMR (400 MHz, $CDCl_3$) δ 10.10 (s, 1H), 9.39 (s, 1H), 7.07 (t, $J$ = 1.6 Hz, 1H), 6.91 (t, $J$ = 1.6 Hz, 1H).

*Preparation of 4-nitro-1H-pyrrole-2-carbaldehyde **1h*** and *5-nitro-1H-pyrrole-2-carbaldehyde **1i***

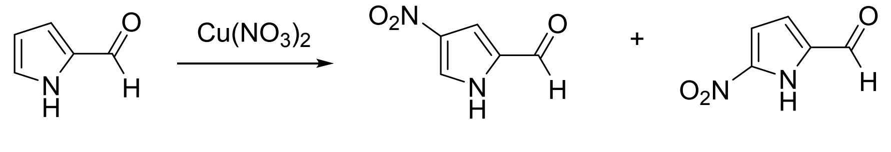


The literature procedure was followed with some modification.[3] The solution of pyrrole-2-carboxaldehyde (0.95g, 1 mmol) in carbon tetrachloride (5 mL) was treated with copper nitrate trihydrate (3.62 g, 1.5 mmol) and acetic anhydride (5.10 g, 5 mmol) and heated at 50 °C. After completion of the reaction (monitored by TLC), the reaction mixture was quenched with $H_2O$ (20 mL) and extracted with EtOAc (20 mL × 2). The organic layer was dried ($Na_2SO_4$) and concentrated. The crude product was purified by column chromatography (hexane/EA = 9/1) to afford **1h** (0.49 g, 35%) and **1i** (0.37 g, 26%).

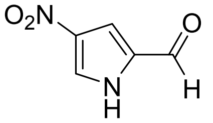

$^1$H NMR (400 MHz, DMSO-$d_6$) δ 13.27 (s, 1H), 9.60 (s, 1H), 8.24 (s, 1H), 7.63 (s, 1H).

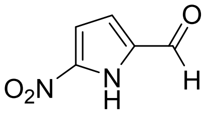

$^1$H NMR (400 MHz, DMSO-$d_6$) δ 14.27 (s, 1H), 9.76 (s, 1H), 7.23 (d, $J$ = 4.0 Hz, 1H), 7.06 (d, $J$ = 4.0 Hz, 1H).

*Preparation of methyl 5-formyl-1H-pyrrole-2-carboxylate **1k***

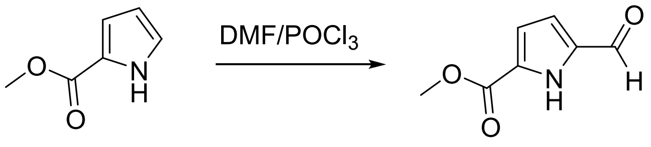


The literature procedure was followed with some modification.[5] $POCl_3$ (2.30 g. 15 mmol) was added to DMF (1.10 g, 15 mmol) for 30 min at 0 °C under nitrogen condition. The solution was diluted with 1, 2-dichloroethane (10 mL) and stirred for 1 h at room temperature. Methyl *1H*-pyrrole-2-carboxylate (1.25 g, 10 mmol) in DCE (10 mL) was added dropwise for 1 h. The reaction mixture was stirred vigorously under reflux condition for 2 h and then neutralized with aq. $NaHCO_3$ (10 mL). After

extraction with DCM (60 mL×5), the combined organic layer was dried over $Na_2SO_4$, filtered, and concentrated in vacuo. Flash column chromatography (hexane/EA = 2:1) afforded a pure compound as a yellow oil. Yield: 1.00 g (65%). $^1$H NMR (400 MHz, $CDCl_3$) δ 9.82 (s, 1H), 9.67 (s, 1H), 6.94 (s, 2H), 3.92 (s, 3H).

*Preparation of 4, 5-dichloro-1H-pyrrole-2-carbaldehyde **1l***

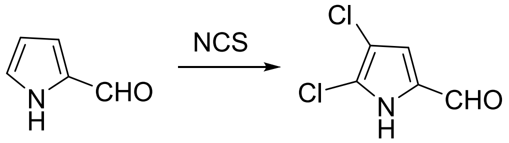


The literature procedure was followed with some modification.[6] To a stirred solution of pyrrole-2-carboxaldehyde (0.95 g, 10 mmol) in acetonitrile (100 mL), NCS (3.34 g, 25 mmol) was added and the resulting solution was stirred 24 h at 50 °C. Then, the solvent was evaporated, and the residue was partitioned between water (100 mL) and DCM (100 mL). The aqueous layer was separated and extracted with DCM (2 × 50 mL). The combined extracts were washed with water (20 mL), dried with $Na_2SO_4$ and the solvents evaporated. The residue was purified by column chromatography (silica gel, hexane/EA = 9:1) affording **1l** as white solid (1.18g, 72 %). $^1$H NMR (400 MHz, $CDCl_3$) δ 10.47 (s, 1H), 9.36 (s, 1H), 6.92 (s, 1H).

**General Procedure for the Preparation of compounds 3a-3l and 5a-5c.**

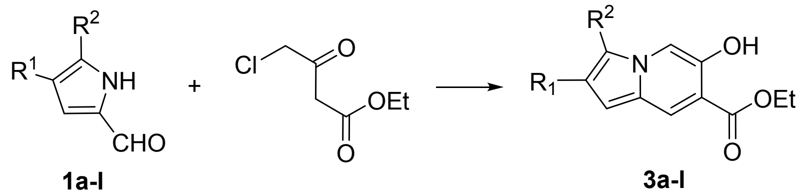


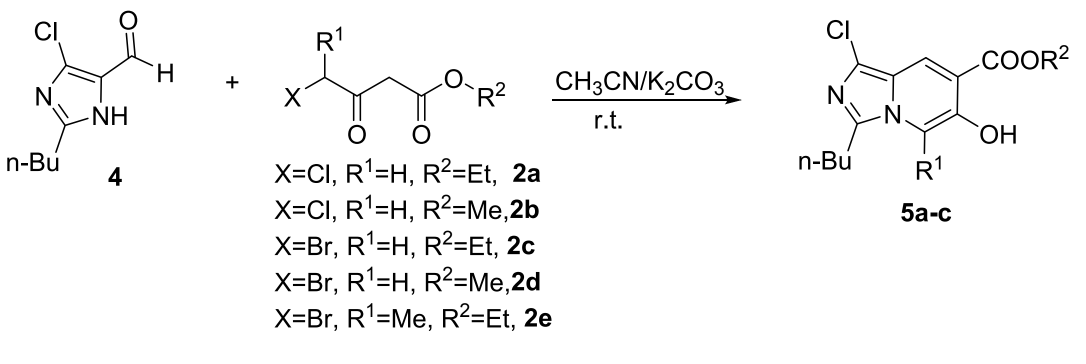


To a stirred solution of substituted pyrrole-2-carboxaldehyde **1** or 2-butyl-4-

chloro-*1H*-imidazole-5-carbaldehyde **4** (1 mmol) in acetonitrile (15 mL), $K_2CO_3$ (0.42 g, 3 mmol) and 4-halogenated acetoacetic ester **2** (2 mmol) were added and the resulting solution was stirred 6 h at r.t. After the reaction was completed, the mixture was poured into water (100 mL) and extracted with dichloromethane (3×30 mL). The combined extracts were washed with water, dried over anhydrous $Na_2SO_4$ and filtered. The solvent was removed by rotary evaporation. The crude products were purified by column chromatography (silica gel, hexane/EA = 20:1) to afford compounds **3a-3l** and **5a-5c**.

*ethyl 6-hydroxy-2-propionylindolizine-7-carboxylate(**3a**)*

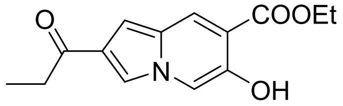


Yellow solid (70% yield). mp: 102-105 °C; $^{1}$H NMR (400 MHz, $CDCl_3$) δ 9.77 (s, 1H), 8.15 (s, 1H), 7.80 (s, 1H), 7.59 (s, 1H), 7.08 (s, 1H), 4.43 (q, *J* = 7.2 Hz, 2H), 2.93 (q, *J* = 7.2 Hz, 2H), 1.45 (t, *J* = 7.2 Hz, 3H), 1.23 (t, *J* = 7.2 Hz, 3H); $^{13}$C NMR (100 MHz, $CDCl_3$) δ 197.3, 168.6, 146.3, 129.5, 128.9, 125.0, 116.8, 110.4, 109.0, 105.6, 61.9, 33.2, 14.2, 8.4; HRMS: m/z calcd for $[M+H]^+$ 262.1079, found 262.1073.

*ethyl 2-acetyl-6-hydroxyindolizine-7-carboxylate (**3b**)*

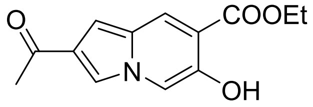


Yellow solid (72% yield). mp: 103-106 °C; $^{1}$H NMR (400 MHz, $CDCl_3$) δ 9.73 (s, 1H), 8.08 (s, 1H), 7.72 (s, 1H), 7.52 (s, 1H), 7.01 (s, 1H), 4.37 (q, *J* = 7.2 Hz, 2H), 2.48 (s, 3H), 1.38 (t, *J* = 7.2 Hz, 3H); $^{13}$C NMR (100 MHz, $CDCl_3$) δ 193.3, 167.5, 145.3, 128.8, 127.9, 124.0, 116.0, 109.5, 107.9, 104.8, 60.9, 28.7, 13.2; HRMS: m/z calcd for $[M+H]^+$ 248.0923, found 248.0918.

*ethyl 2-benzoyl-6-hydroxyindolizine-7-carboxylate (**3c**)*

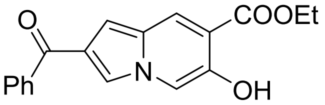


Yellow solid (65% yield). mp: 107-110 °C; $^{1}$H NMR (400 MHz, $CDCl_3$) δ 9.72 (s, 1H), 8.11 (s, 1H), 7.84 (d, $J$ = 8.0 Hz, 2H), 7.74 (s, 1H), 7.54 (s, 1H), 7.51 (d, $J$ = 6.8 Hz, 2H), 7.44 (t, $J$ = 7.2 Hz, 2H), 7.06 (s, 1H), 4.37 (q, $J$ = 7.2 Hz, 2H), 1.38 (t, $J$ = 7.2 Hz, 3H); $^{13}$C NMR (100 MHz, $CDCl_3$) δ 191.2, 168.6, 146.4, 139.1, 132.1, 129.3, 128.8, 128.6, 128.4, 125.1, 118.8, 110.6, 108.9, 107.8, 61.9, 14.2; HRMS: m/z calcd for $[M+H]^+$ 310.1079, found 310.1077.

*7-ethyl 2-methyl 6-hydroxyindolizine-2,7-dicarboxylate (**3d**)*

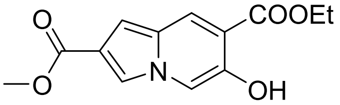


Yellow solid (69% yield). mp: 105-108 °C; $^{1}$H NMR (400 MHz, $CDCl_3$) δ 9.76 (s, 1H), 8.13 (s, 1H), 7.80(s, 1H), 7.59 (s, 1H), 7.11 (s, 1H), 4.43 (q, $J$ =7.2 Hz, 2H), 3.88 (s, 3H), 1.44 (t, $J$ = 6.8 Hz, 3H); $^{13}$C NMR (100 MHz, $CDCl_3$) δ 168.6, 164.8, 146.1, 128.6, 124.7, 121.2, 117.8, 110.2, 108.9, 106.8, 61.9, 51.6, 14.2; HRMS: m/z calcd for $[M+H]^+$ 264.0872, found 264.0868.

*ethyl 2-formyl-6-hydroxyindolizine-7-carboxylate (**3e**)*

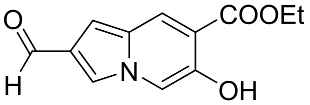


Yellow solid (75% yield). mp: 100-103 °C; $^{1}$H NMR (400 MHz, $CDCl_3$) δ 10.03 (s, 1H), 9.81 (s, 1H), 8.19 (s, 1H), 7.82 (s, 1H), 7.62 (s, 1H), 7.12 (s, 1H), 4.44 (q, $J$ = 7.2 Hz, 2H), 1.45 (t, $J$ = 6.8 Hz, 3H); $^{13}$C NMR (100 MHz, $CDCl_3$) δ 186.7, 168.5, 146.6, 129.6, 129.4, 125.3, 117.9, 110.1, 109.2, 106.1, 62.0, 14.2; HRMS: m/z calcd for $[M+H]^+$ 234.0766, found 234.0761.

*ethyl 2-bromo-6-hydroxyindolizine-7-carboxylate* ***(3f)***

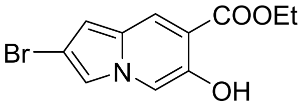


Yellow solid (60% yield). mp: 110-113 °C; $^{1}H$ NMR (400 MHz, $CDCl_3$) δ 9.72 (s, 1H), 8.02(s, 1H), 7.58 (s, 1H), 7.34 (s, 1H), 6.70 (s, 1H), 4.42 (q, $J$ = 7.2 Hz, 2H), 1.44 (t, $J$ = 7.2 Hz, 3H); $^{13}C$ NMR (100 MHz, $CDCl_3$) δ 168.5, 145.5, 128.8, 121.8, 115.2, 109.1, 108.6, 106.5, 104.3, 61.7, 14.2; HRMS: m/z calcd for $[M]^+$ 282.9844, found 282.2790.

*ethyl 6-hydroxy-2-iodoindolizine-7-carboxylate* ***(3g)***

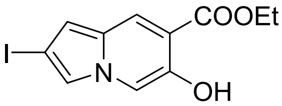


Yellow solid (55% yield). mp: 106-108 °C; $^{1}H$ NMR (400 MHz, $CDCl_3$) δ 9.70 (s, 1H), 8.02 (s, 1H), 7.58 (s, 1H), 7.38 (s, 1H), 6.81 (s, 1H), 4.42 (q, $J$ = 6.8 Hz, 2H), 1.43 (t, $J$ = 6.8 Hz, 3H); $^{13}C$ NMR (100 MHz, $CDCl_3$) δ 168.5, 145.4, 130.2, 121.4, 119.8, 112.0, 109.0, 108.2, 61.7, 14.2; HRMS: m/z calcd for $[M+H]^+$ 330.9705, found 330.9700.

*ethyl 6-hydroxy-2-nitroindolizine-7-carboxylate* ***(3h)***

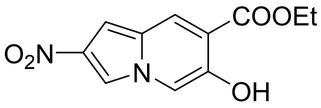


Yellow solid (74% yield). mp: 135-137 °C; $^{1}H$ NMR (400 MHz, $CDCl_3$) δ 9.82 (s, 1H), 8.10 (s, 1H), 7.92 (s, 1H), 7.54 (s, 1H), 7.15 (s, 1H), 4.39 (q, $J$ = 7.2 Hz, 2H), 1.39 (t, $J$ = 7.2 Hz, 3H); $^{13}C$ NMR (100 MHz, $CDCl_3$) δ 167.1, 146.2, 126.2, 124.2, 113.3, 111.4, 108.4, 99.8, 61.3, 13.1; HRMS: m/z calcd for $[M+H]^+$ 251.0668, found 251.0663. HRMS: m/z calcd for $[M+H]^+$ 251.0668, found 251.0663.

*ethyl 6-hydroxy-3-nitroindolizine-7-carboxylate (**3i**)*

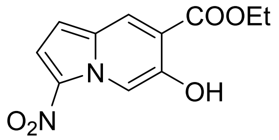


Yellow solid (66% yield). mp: 136-140 °C; $^1H$ NMR (400 MHz, $CDCl_3$) δ 10.09 (s, 1H), 9.44 (s, 1H), 8.24 (s, 1H), 7.73 (d, $J$ = 5.2 Hz, 1H), 6.76 (d, $J$ = 5.2 Hz, 1H), 4.51 (q, $J$ = 7.2 Hz, 2H), 1.48 (t, $J$ = 6.8 Hz, 3H); $^{13}C$ NMR (100 MHz, $CDCl_3$) δ 167.8, 149.9, 132.3, 121.5, 118.1, 114.3, 107.0, 62.8, 14.2;

*diethyl 6-hydroxyindolizine-3,7-dicarboxylate (**3j**)*

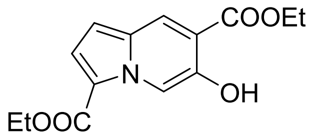


Yellow solid (66% yield). mp: 122-125 °C; $^1H$ NMR (400 MHz, $CDCl_3$) δ 9.77 (s, 1H), 9.20 (s, 1H), 8.16 (s, 1H), 7.46 (d, $J$ = 4.0 Hz, 1H), 6.68 (d, $J$ = 4.0 Hz, 1H), 4.46 (q, $J$ = 7.2 Hz, 2H), 4.40 (q, $J$ = 6.8 Hz, 2H), 1.45 (t, $J$ = 7.2Hz, 3H), 1.41 (t, $J$ = 6.8 Hz, 3H); $^{13}C$ NMR (100 MHz, $CDCl_3$) δ 168.6, 161.1, 146.9, 132.9, 121.5, 116.5, 112.9, 111.4, 105.4, 62.0, 60.2, 14.6, 14.2; HRMS: m/z calcd for $[M+H]^+$ 278.1028, found 278.1025.

*7-ethyl 3-methyl 6-hydroxyindolizine-3,7-dicarboxylate (**3k**)*

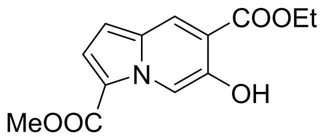


Yellow solid (70% yield). mp: 107-109 °C; $^1H$ NMR (400 MHz, $CDCl_3$) δ 9.70 (s, 1H), 9.12 (s, 1H), 8.09 (s, 1H), 7.37 (d, $J$ = 4.8 Hz, 1H), 6.60 (d, $J$ = 4.8 Hz, 1H), 4.34 (q, $J$ = 7.2 Hz, 2H), 3.85 (s, 3H), 1.38 (t, $J$ = 7.2 Hz, 3H); $^{13}C$ NMR (100 MHz, $CDCl_3$) δ 167.5, 160.4, 146.0, 131.9, 120.5, 120.4, 115.1, 111.9, 110.5, 104.5, 61.0, 50.3, 13.2; HRMS: m/z calcd for $[M+H]^+$ 264.0872, found 264.0867.

*ethyl 2,3-dichloro-6-hydroxyindolizine-7-carboxylate (**3l**)*

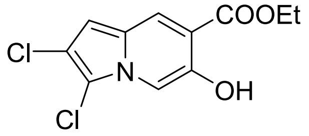


Yellow solid (62% yield). mp: 100-104 °C; $^{1}$H NMR (400 MHz, $CDCl_3$) δ 9.88 (s, 1H), 8.02 (s, 1H), 7.61 (s, 1H), 6.73 (s, 1H), 4.43 (q, $J$ = 7.2 Hz, 2H), 1.44 (t, $J$ = 7.2 Hz, 3H); $^{13}$C NMR (100 MHz, $CDCl_3$) δ 168.5, 146.3, 126.2, 121.9, 118.5, 106.2, 103.6, 62.1, 14.2; HRMS: m/z calcd for $[M+H]^+$ 274.0038, found 274.2740.

*ethyl 3-butyl-1-chloro-6-hydroxyimidazo[1,5-a]pyridine-7-carboxylate (**5a**)*

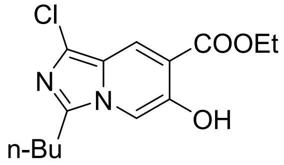


Yellow solid (2a: 63% yield; 2e: 68% yield). mp: 116-119 °C; $^{1}$H NMR (400 MHz, $CDCl_3$) δ 9.90 (s, 1H), 8.14 (s, 1H), 7.34 (s, 1H), 4.45 (q, $J$ = 7.2 Hz, 2H), 2.88 (t, $J$ = 7.6 Hz, 2H), 1.76-1.83(m, 2H), 1.40-1.47(m, 5H), 0.96 (t, $J$ = 7.6 Hz, 3H); $^{13}$C NMR (100 MHz, $CDCl_3$) δ 168.3, 146.4, 138.9, 124.4, 122.3, 122.0, 110.0, 103.8, 62.2, 28.7, 26.4, 22.5, 14.2, 13.8; HRMS: m/z calcd for $[M+H]^+$ 297.1006, found 297.1010.

*methyl 3-butyl-1-chloro-6-hydroxyimidazo[1,5-a]pyridine-7-carboxylate (**5b**)*

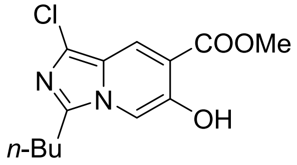


Yellow solid (2b: 68% yield; 2d: 72% yield). mp: 87-90 °C; $^{1}$H NMR (400 MHz, $CDCl_3$) δ 9.73 (s, 1H), 8.09 (s, 1H), 7.27 (s, 1H), 3.92 (s, 3H), 2.81 (t, $J$ = 8.0 Hz, 2H), 1.69-1.76 (m, 2H), 1.31-1.40 (m, 2H), 0.89 (t, $J$ = 7.2 Hz, 3H); $^{13}$C NMR (100 MHz, $CDCl_3$) δ 167.8, 145.2, 138.0, 123.7, 121.4, 121.0, 108.6, 102.8, 51.8, 27.6, 25.4, 21.5, 12.8; HRMS: m/z calcd for $[M+H]^+$ 283.0849, found 283.0852.

*ethyl 3-butyl-1-chloro-6-hydroxy-5-methylimidazo[1,5-a]pyridine-7-carboxylate (**5c**)*

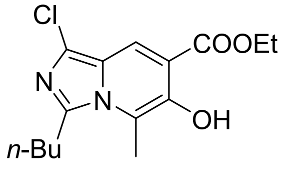


Yellow solid (66% yield). mp: 70-73 °C; $^{1}$H NMR (400 MHz, $CDCl_3$) δ 9.93 (s, 1H), 7.94 (s, 1H), 4.36 (q, *J* = 7.2 Hz, 2H), 3.18 (t, *J* = 7.6 Hz, 3H), 2.64 (s, 3H), 1.69-1.76 (m, 2H), 1.33-1.39 (m, 5H), 0.89 (q, *J* = 7.2 Hz, 3H); $^{13}$C NMR (100 MHz, $CDCl_3$) δ 167.6, 141.4, 140.1, 123.3, 122.0, 118.9, 114.6, 108.7, 61.1, 31.3, 29.1, 21.4, 13.2, 12.8, 10.8; HRMS: m/z calcd for $[M+H]^+$ 311.1162, found 311.1171.

## Reference


1. G. H. Barnett, H. J. Anderson and C. E. Loader, *Can. J. Chem.*, 1980, **58**, 409-411.
2. H. J. Anderson, C. E. Loader and A. Foster, *Can. J. Chem.*, 1980, **58**, 2527-2530.
3. V. K. Outlaw, F. B. d'Andrea and C. A. Townsend, *Org. Lett.*, 2015, **17**, 1822-1825.
4. J. K. Laha, S. Sharma, S. Kirar and U. C. Banerjee, *J. Org. Chem.*, 2017, **82**, 9350-9359.
5. H. H. Lee, J. B. Lee, Y. Park, K. H. Park, M. S. Okyay, D. S. Shin, S. Kim, J. Park, N. Park, B. K. An, Y. S. Jung, H. W. Lee, K. T. Lee and S. Y., *ACS Appl. Mater. Interfaces*, 2018, **10**, 22110-22118.
6. C. Santiago, I. Rubio, N. Sotomayor and E. Lete, *Eur. J. Org. Chem.* 2020, 4284-4295.

**Table S1** The optical characteristics of the compounds **3a-l** in EtOH

| compounds | $\lambda_{max}$(nm) | $\lambda_1$ (nm) | $\lambda_2$ (nm) | $\lambda_{em}$ (nm) |
|---|---|---|---|---|
| **3a** | 257 | 290 | 395 | 540 |
| **3b** | 256 | 293 | 390 | 535 |
| **3c** | 252 | 310 | 415 | 450 |
| **3d** | 262 | 350 | 395 | 505 |
| **3e** | 254 | 340 | 405 | 425 |
| **3f** | 242 | 352 | 390 | 450 |
| **3g** | 243 | 353 | 395 | 450 |
| **3h** | 248 | 320 | 410 | 450 |
| **3i** | 242 | 320 | 445 | 455 |
| **3j** | 250 | 290 | 380 | 455 |
| **3k** | 236 | 290 | 385 | 455 |
| **3l** | 260 | 355 | 385 | 450 |

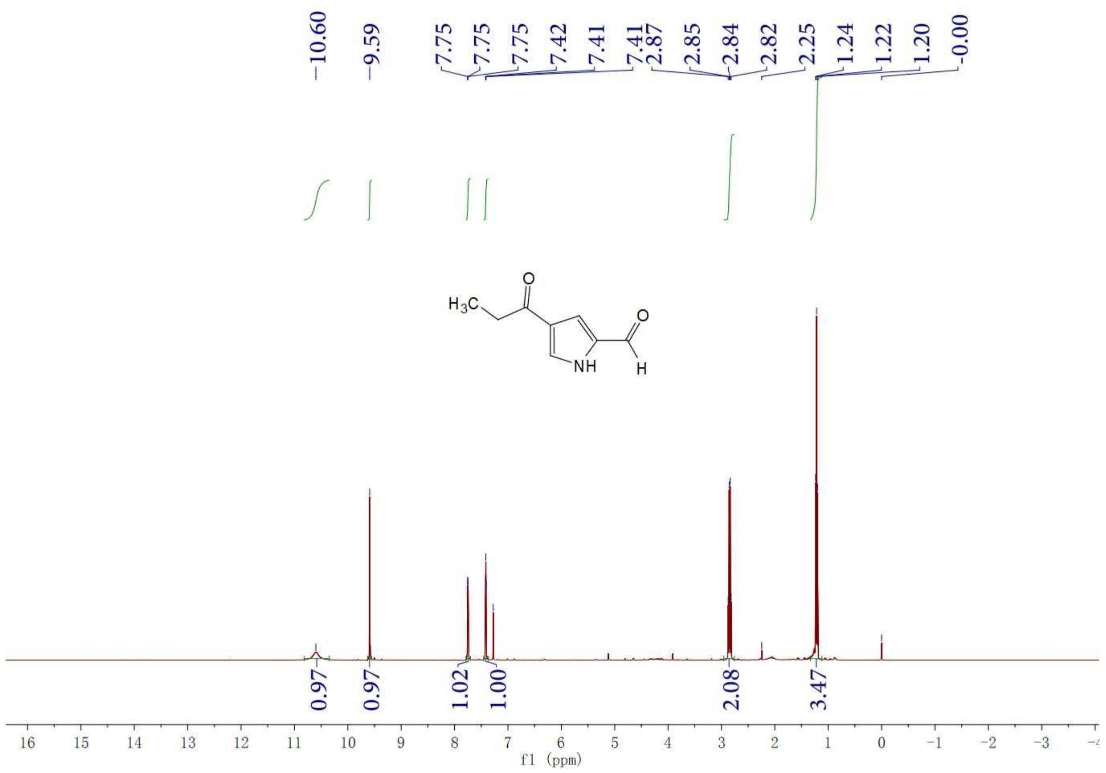

10.60
9.59
7.75
7.75
7.75
7.42
7.41
7.41
2.87
2.85
2.84
2.82
2.25
1.24
1.22
1.20
-0.00
H3C
O
O
NH
H
0.97
0.97
1.02
1.00
2.08
3.47
16 15 14 13 12 11 10 9 8 7 6 5 4 3 2 1 0 −1 −2 −3
f1 (ppm)


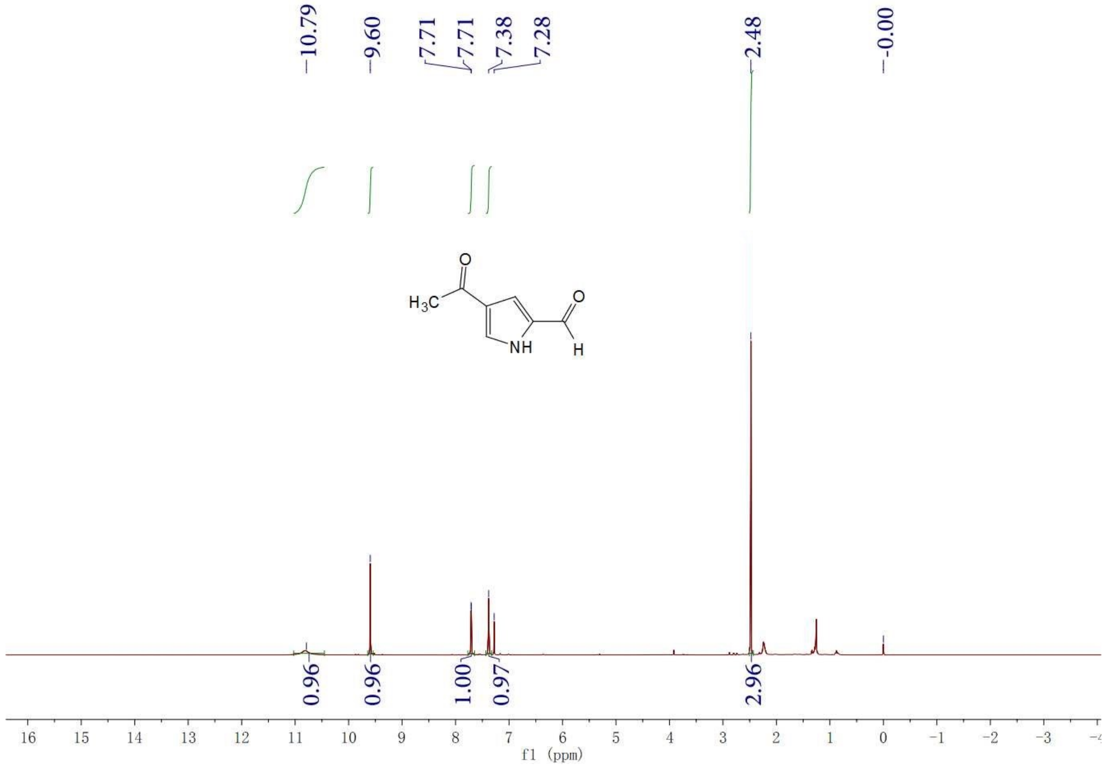

10.79
9.60
7.71
7.71
7.38
7.28
2.48
-0.00
H3C
O
O
NH
H
0.96
0.96
1.00
0.97
2.96
16 15 14 13 12 11 10 9 8 7 6 5 4 3 2 1 0 −1 −2 −3
f1 (ppm)

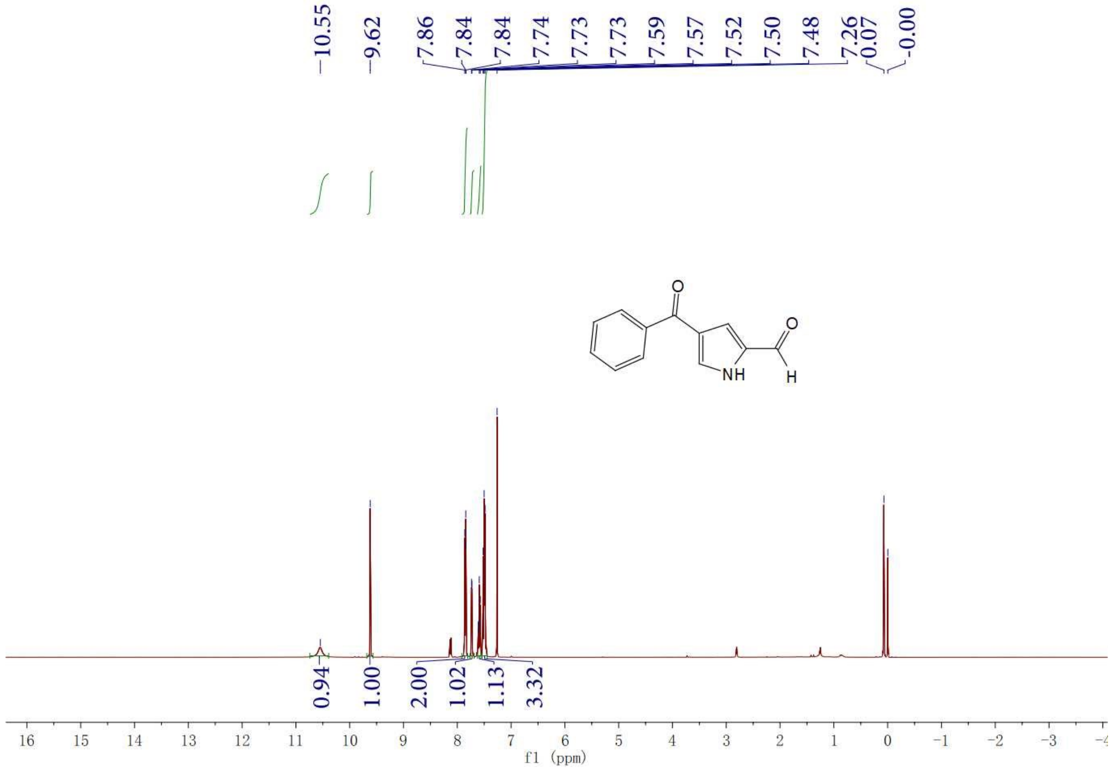

10.55
9.62
7.86
7.84
7.84
7.74
7.73
7.73
7.59
7.57
7.52
7.50
7.48
7.26
0.07
-0.00
O
O
NH
H
0.94
1.00
2.00
1.02
1.13
3.32
16 15 14 13 12 11 10 9 8 7 6 5 4 3 2 1 0 −1 −2 −3
f1 (ppm)


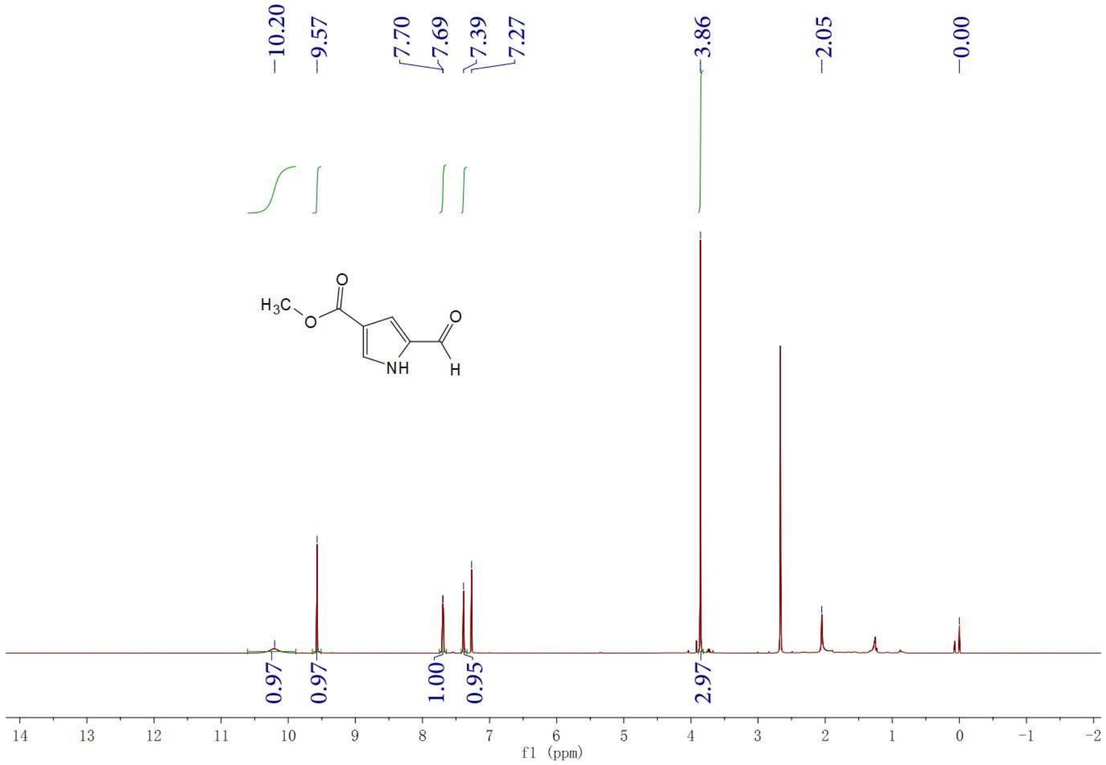

10.20
9.57
7.70
7.69
7.39
7.27
3.86
2.05
0.00
$H_3C$
O
O
O
NH
H
0.97
0.97
1.00
0.95
2.97
14 13 12 11 10 9 8 7 6 5 4 3 2 1 0 −1 −2
f1 (ppm)

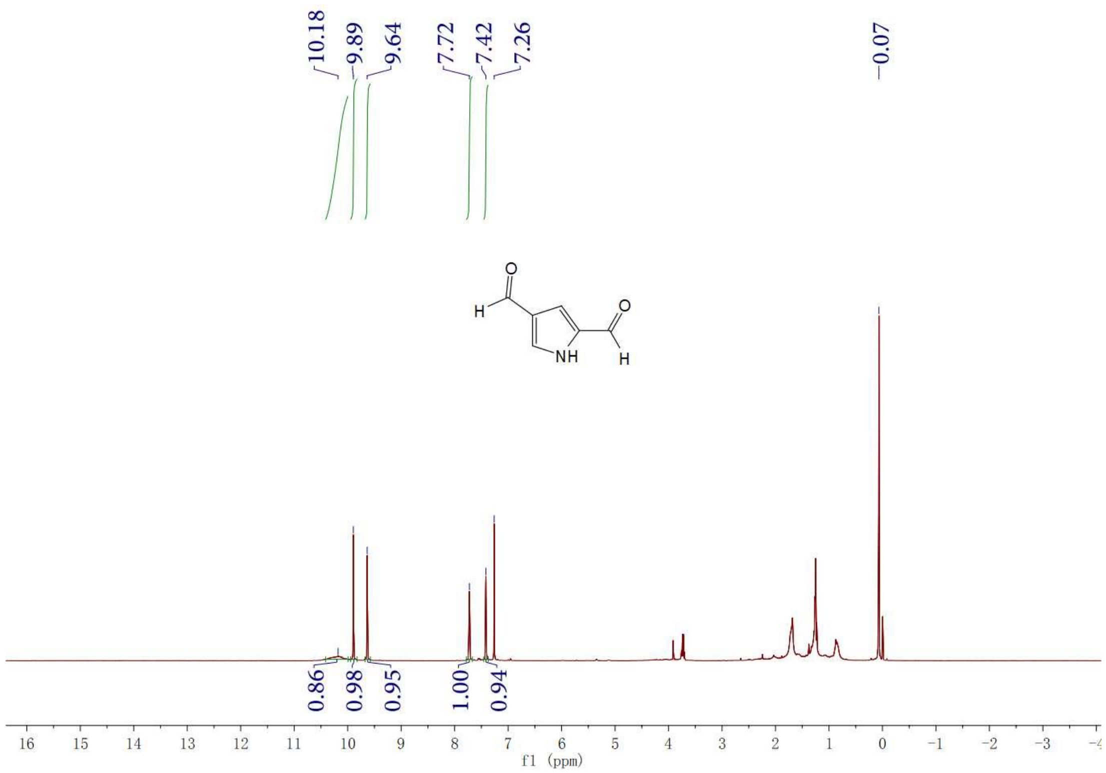
10.18
9.89
9.64
7.72
7.42
7.26
-0.07
H
O
O
NH
H
0.86
0.98
0.95
1.00
0.94
16 15 14 13 12 11 10 9 8 7 6 5 4 3 2 1 0 -1 -2 -3
f1 (ppm)

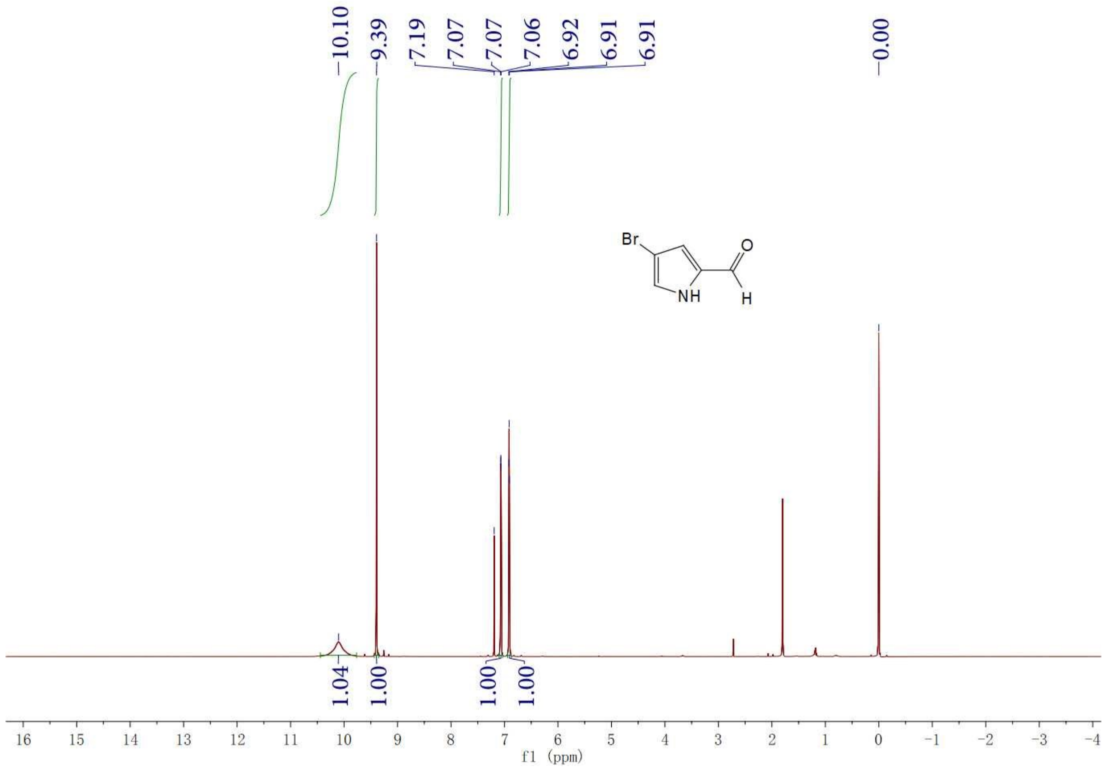
10.10
9.39
7.19
7.07
7.07
7.06
6.92
6.91
6.91
0.00
Br
O
NH
H
1.04
1.00
1.00
1.00
16 15 14 13 12 11 10 9 8 7 6 5 4 3 2 1 0 -1 -2 -3 -4
f1 (ppm)

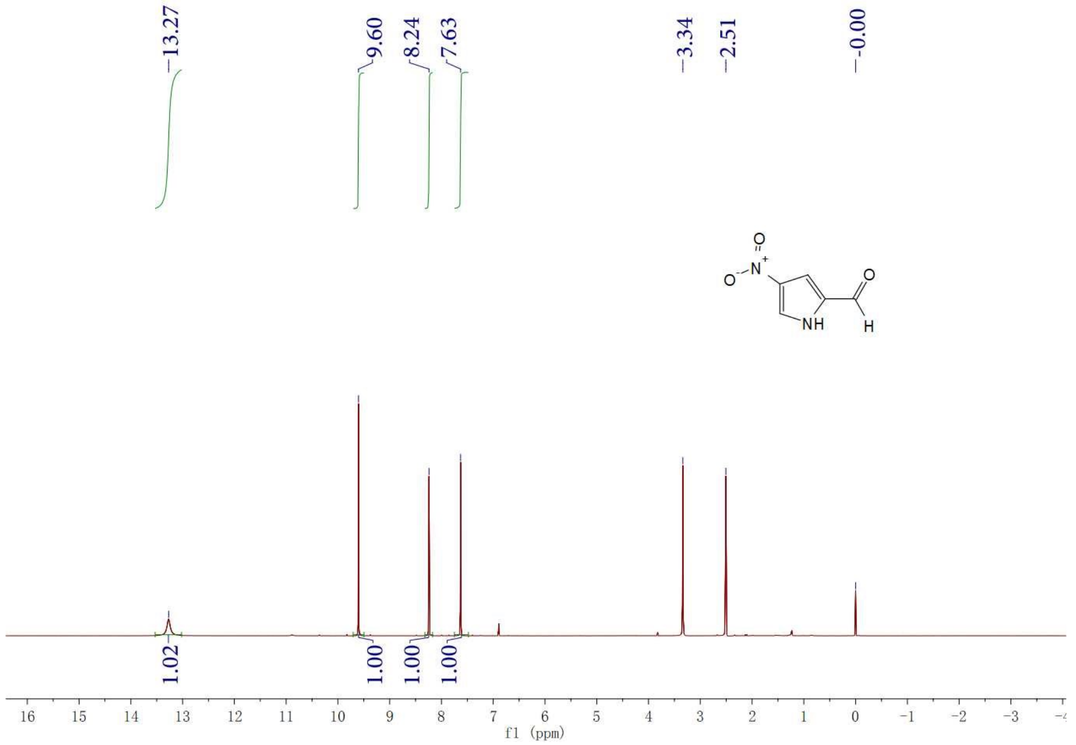
13.27
9.60
8.24
7.63
3.34
2.51
-0.00
1.02
1.00
1.00
1.00
f1 (ppm)
NH
H

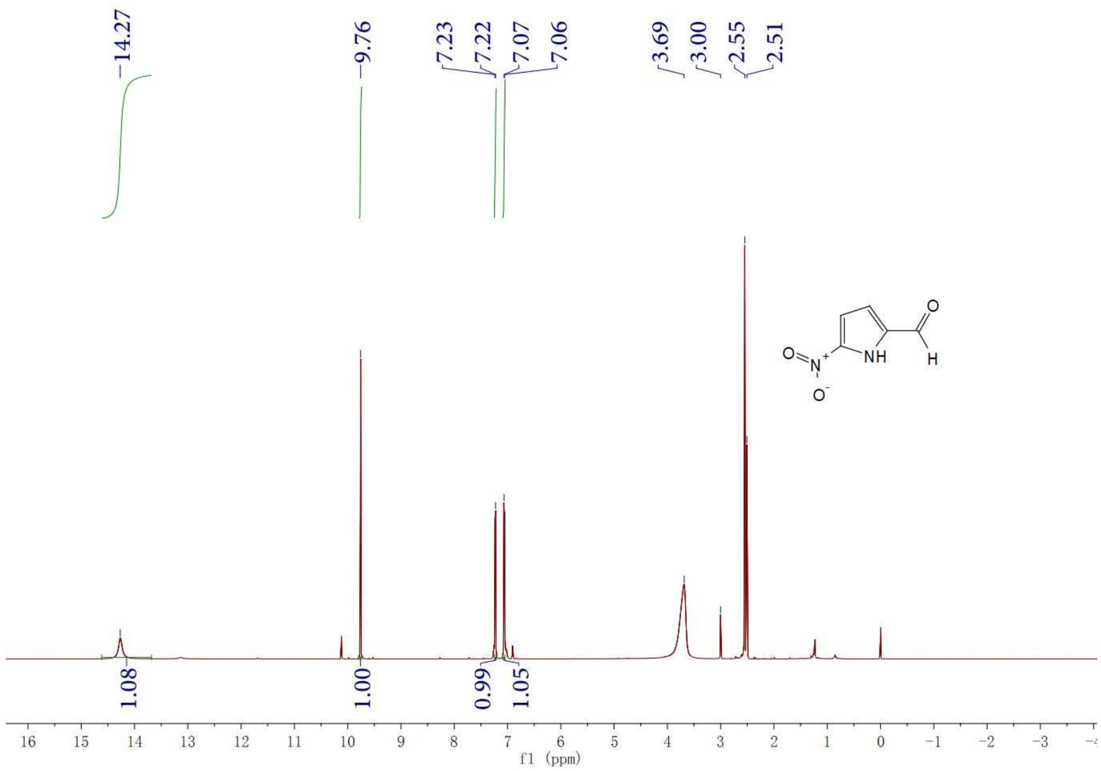
14.27
9.76
7.23
7.22
7.07
7.06
3.69
3.00
2.55
2.51
1.08
1.00
0.99
1.05
f1 (ppm)
NH
H

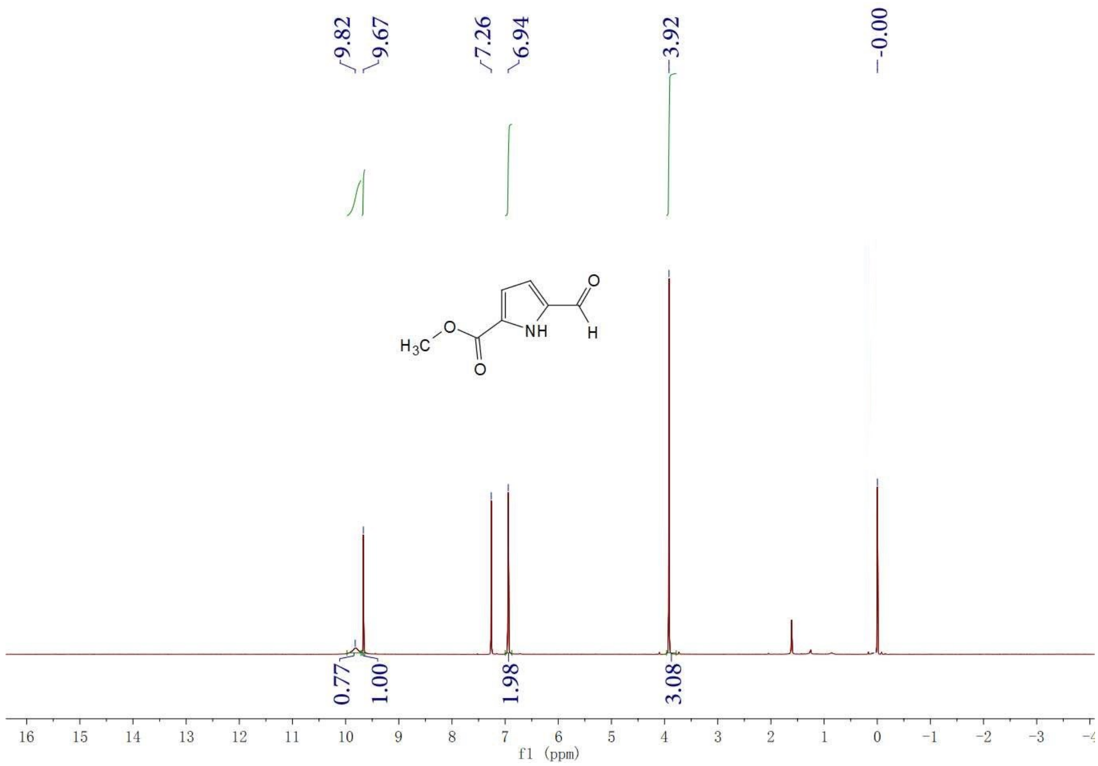
9.82
9.67
7.26
6.94
3.92
-0.00
H3C
O
O
NH
O
H
0.77
1.00
1.98
3.08
16 15 14 13 12 11 10 9 8 7 6 5 4 3 2 1 0 -1 -2 -3
f1 (ppm)

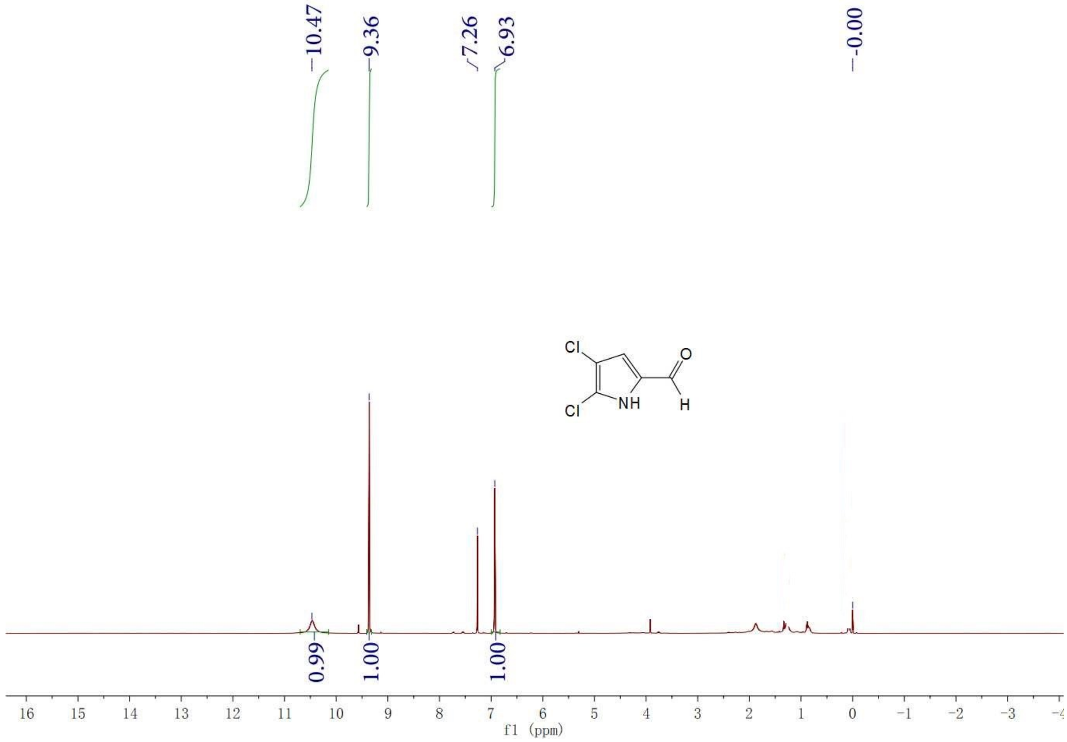
10.47
9.36
7.26
6.93
-0.00
Cl
Cl
NH
O
H
0.99
1.00
1.00
16 15 14 13 12 11 10 9 8 7 6 5 4 3 2 1 0 -1 -2 -3
f1 (ppm)

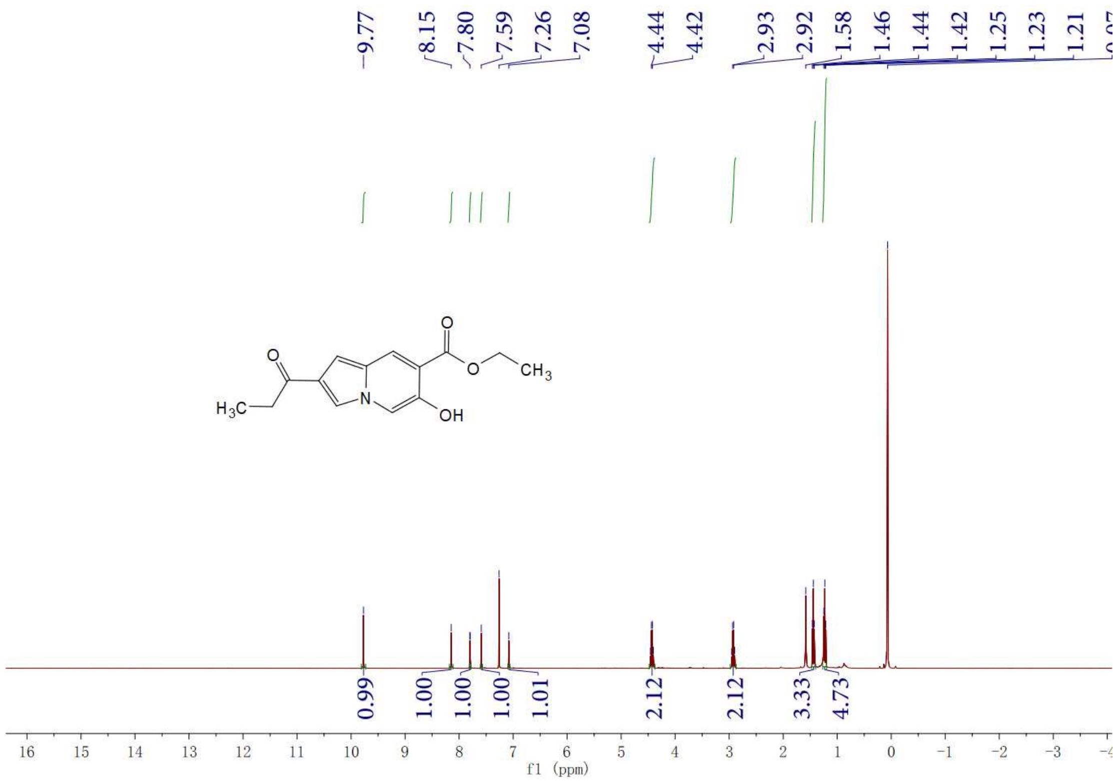
9.77
8.15
7.80
7.59
7.26
7.08
4.44
4.42
2.93
2.92
1.58
1.46
1.44
1.42
1.25
1.23
1.21
0.99
1.00
1.00
1.00
1.01
2.12
2.12
3.33
4.73
OH
CH3
H3C
f1 (ppm)

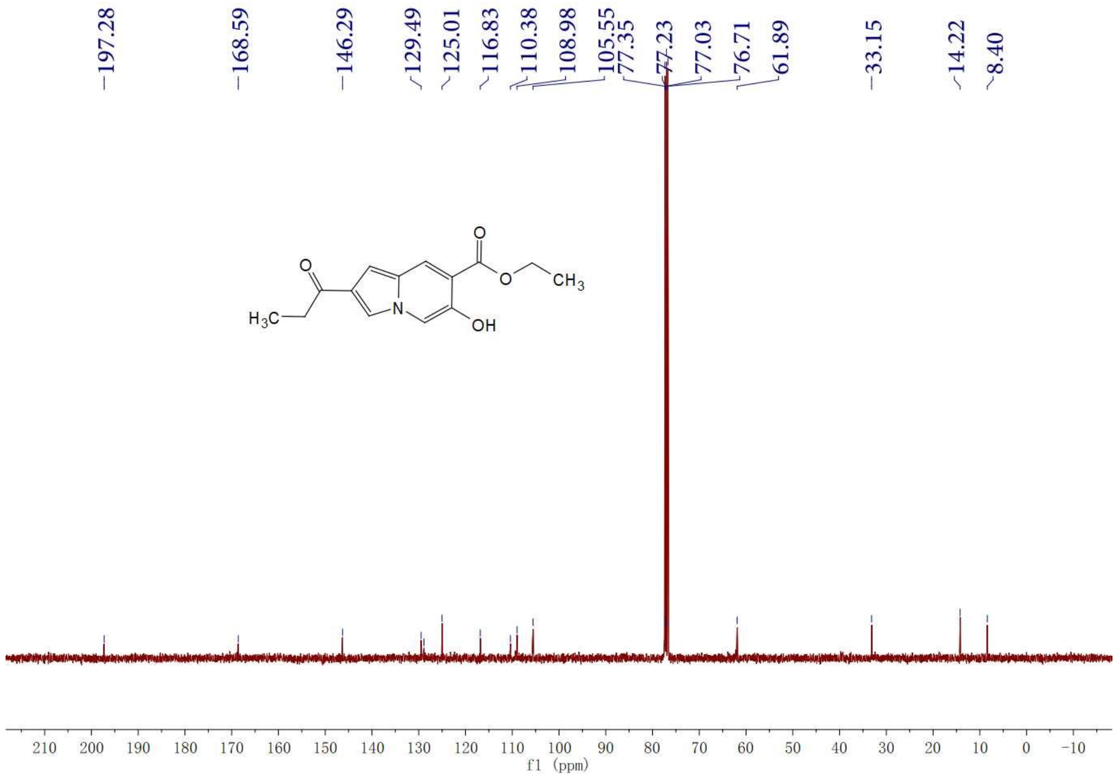
197.28
168.59
146.29
129.49
125.01
116.83
110.38
108.98
105.55
77.35
77.23
77.03
76.71
61.89
33.15
14.22
8.40
OH
CH3
H3C
f1 (ppm)

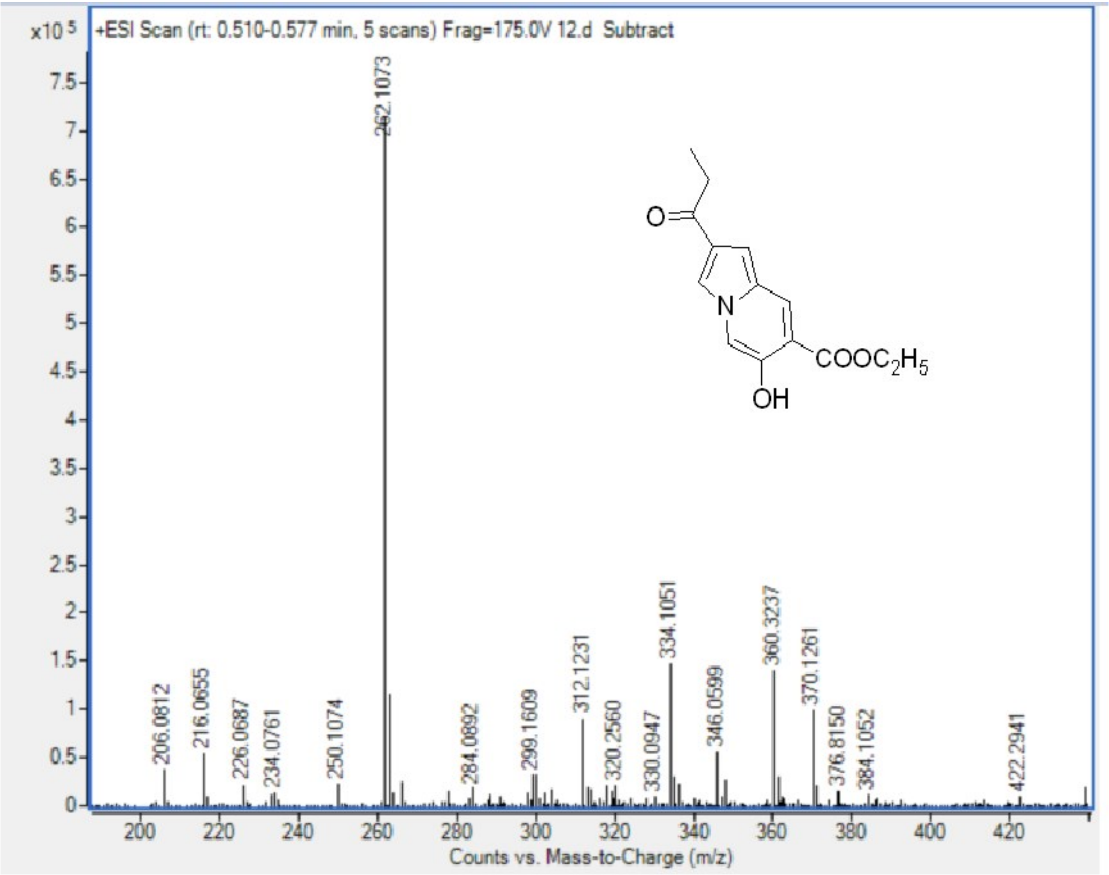
x10 5
+ESI Scan (rt: 0.510-0.577 min, 5 scans) Frag=175.0V 12.d Subtract
262.1073
206.0812
216.0655
226.0687
234.0761
250.1074
284.0892
299.1609
312.1231
320.2560
330.0947
334.1051
346.0599
360.3237
370.1261
376.8150
384.1052
422.2941
Counts vs. Mass-to-Charge (m/z)
O
N
COOC2H5
OH

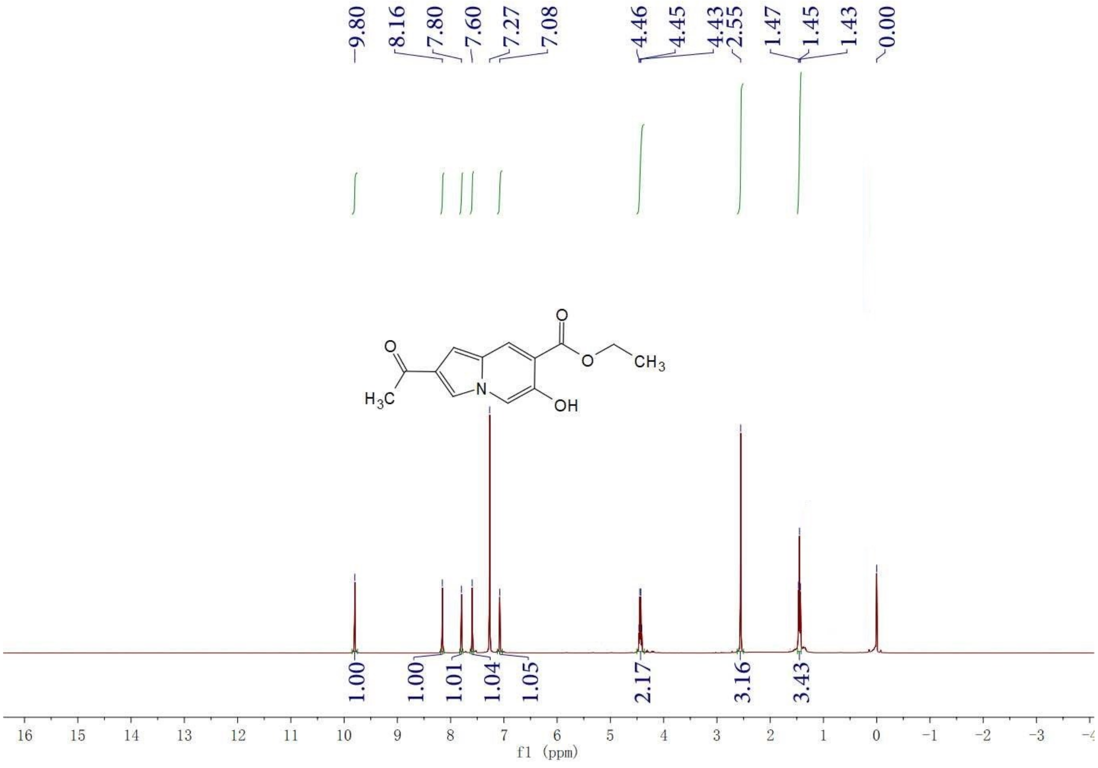
9.80
8.16
7.80
7.60
7.27
7.08
4.46
4.45
4.43
2.55
1.47
1.45
1.43
0.00
O
O
CH3
N
H3C
OH
1.00
1.00
1.01
1.04
1.05
2.17
3.16
3.43
f1 (ppm)

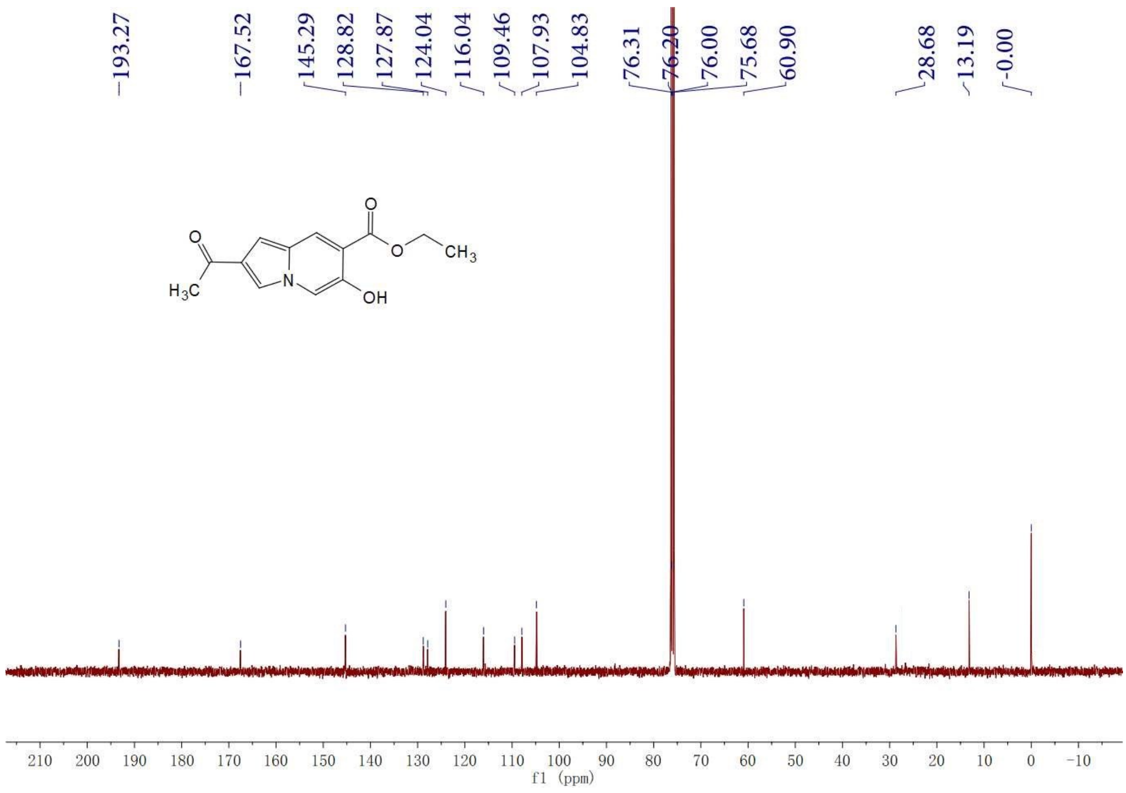
193.27
167.52
145.29
128.82
127.87
124.04
116.04
109.46
107.93
104.83
76.31
76.20
76.00
75.68
60.90
28.68
13.19
-0.00
O
O
O
CH3
H3C
N
OH
210 200 190 180 170 160 150 140 130 120 110 100 90 80 70 60 50 40 30 20 10 0 -10
f1 (ppm)

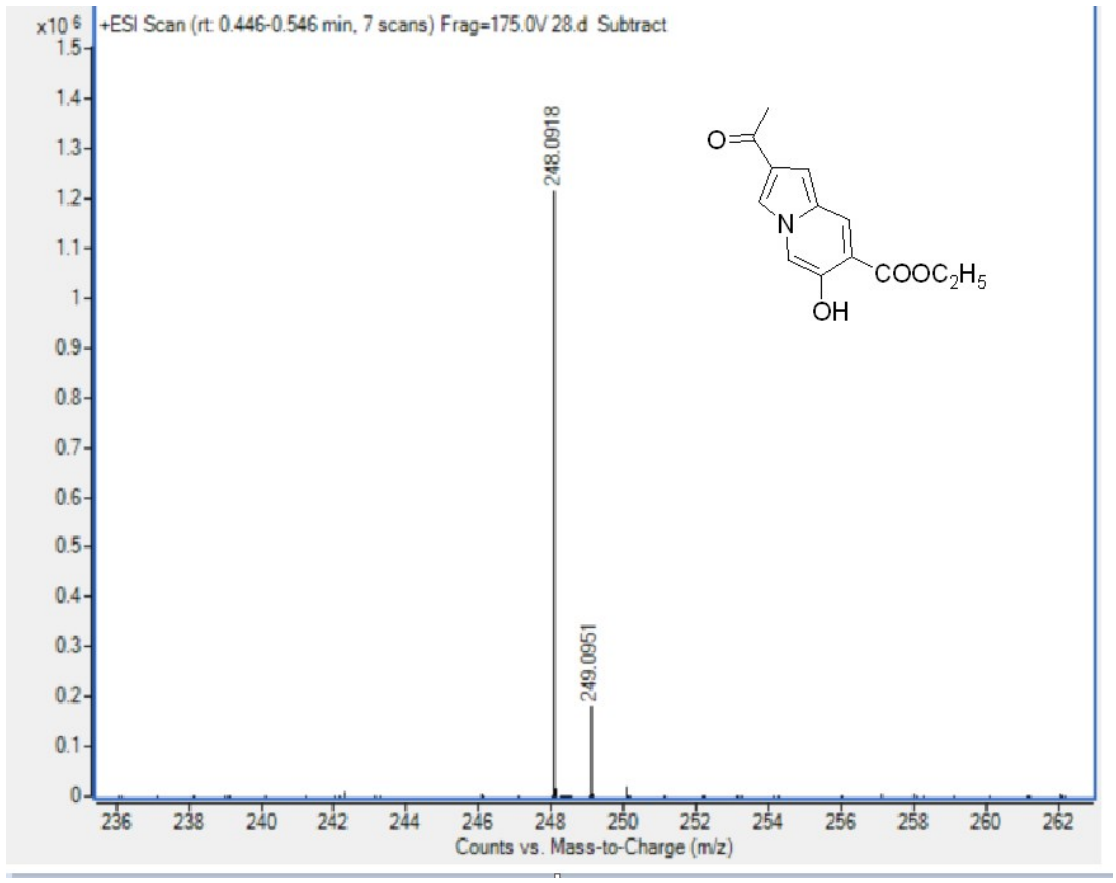
x10 6
+ESI Scan (rt: 0.446-0.546 min, 7 scans) Frag=175.0V 28.d Subtract
248.0918
249.0951
O
N
COOC2H5
OH
Counts vs. Mass-to-Charge (m/z)

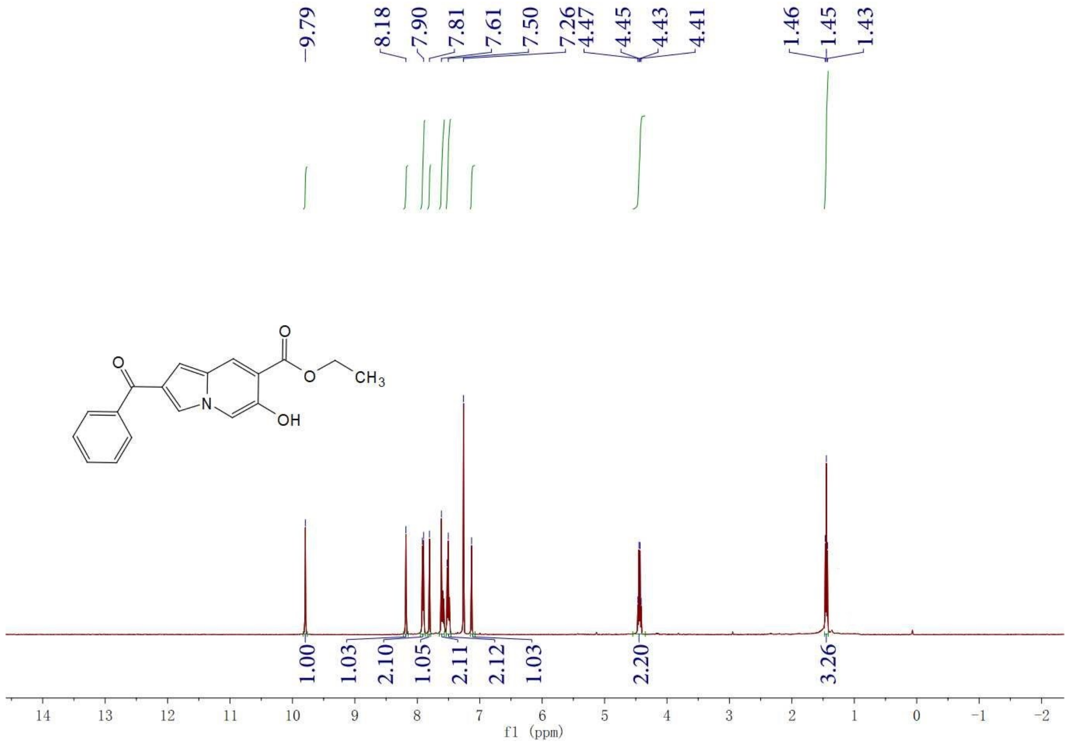
9.79
8.18
7.90
7.81
7.61
7.50
7.26
4.47
4.45
4.43
4.41
1.46
1.45
1.43
O
O
O
CH3
N
OH
1.00
1.03
2.10
1.05
2.11
2.12
1.03
2.20
3.26
14 13 12 11 10 9 8 7 6 5 4 3 2 1 0 −1 −2
f1 (ppm)

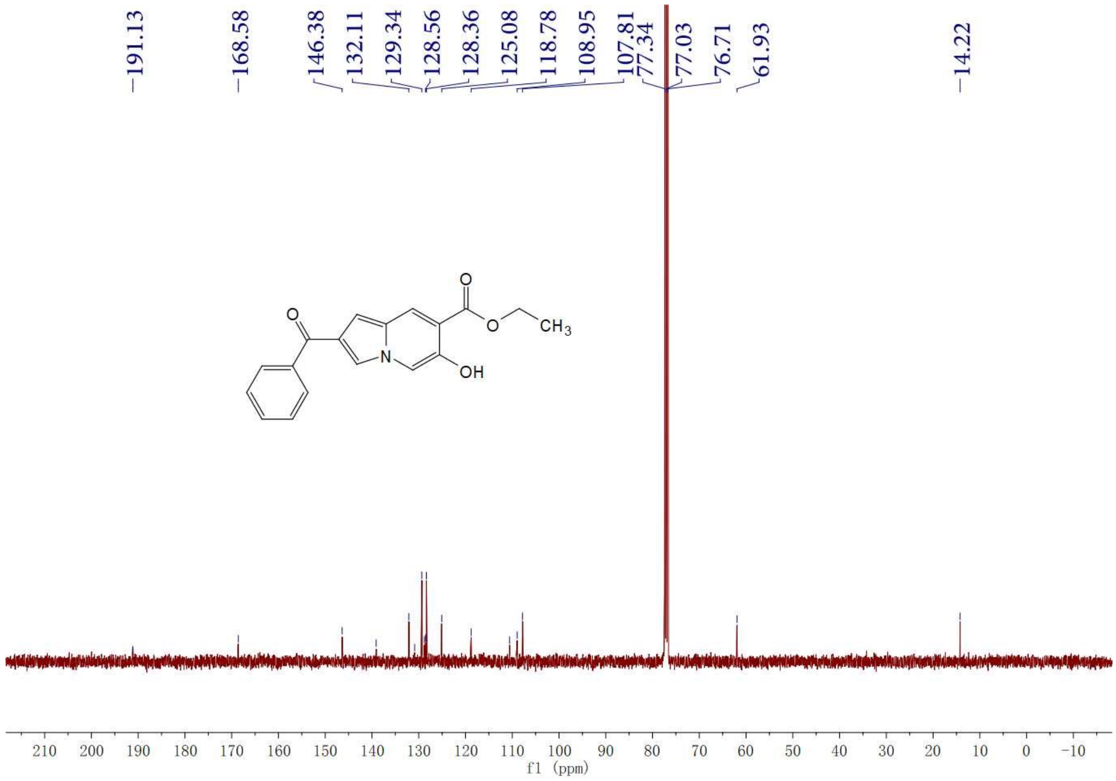
191.13
168.58
146.38
132.11
129.34
128.56
128.36
125.08
118.78
108.95
107.81
77.34
77.03
76.71
61.93
14.22
O
O
O
CH3
N
OH
210 200 190 180 170 160 150 140 130 120 110 100 90 80 70 60 50 40 30 20 10 0 −10
f1 (ppm)

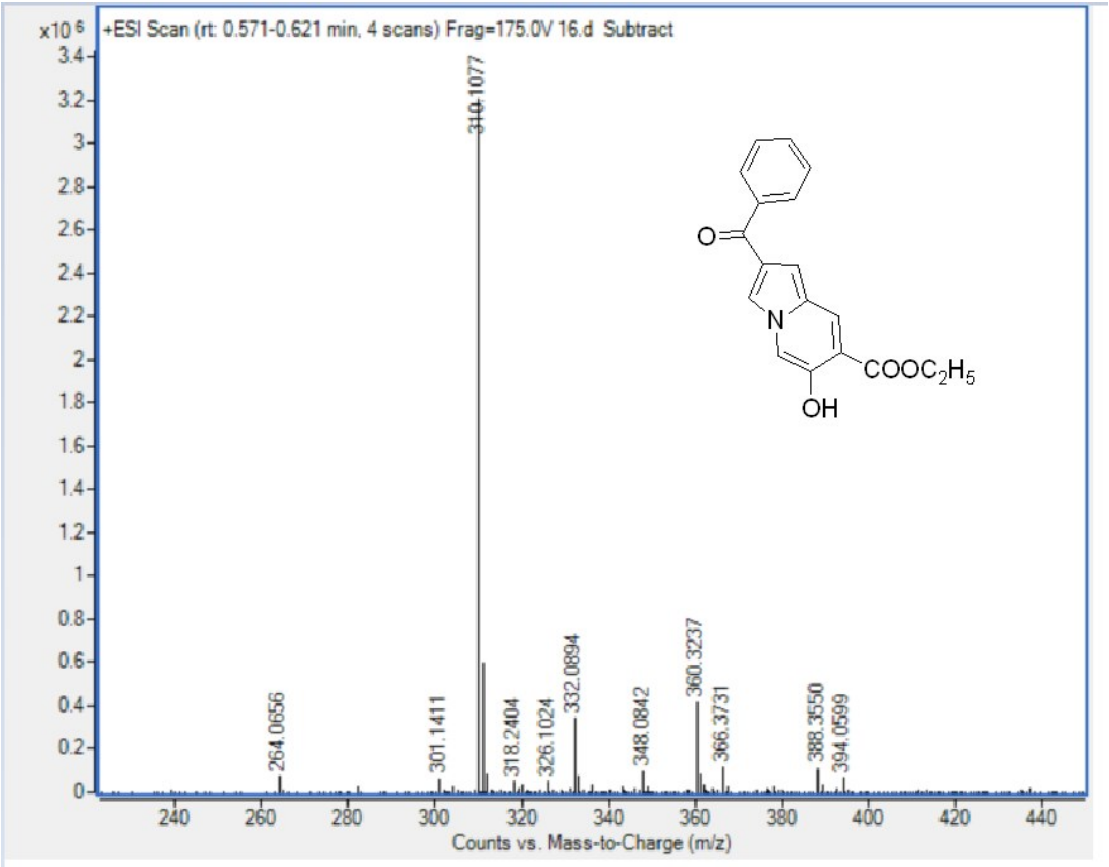
x10 6
+ESI Scan (rt: 0.571-0.621 min, 4 scans) Frag=175.0V 16.d Subtract
310.1077
264.0656
301.1411
318.2404
326.1024
332.0894
348.0842
360.3237
366.3731
388.3550
394.0599
O
N
COOC2H5
OH
Counts vs. Mass-to-Charge (m/z)

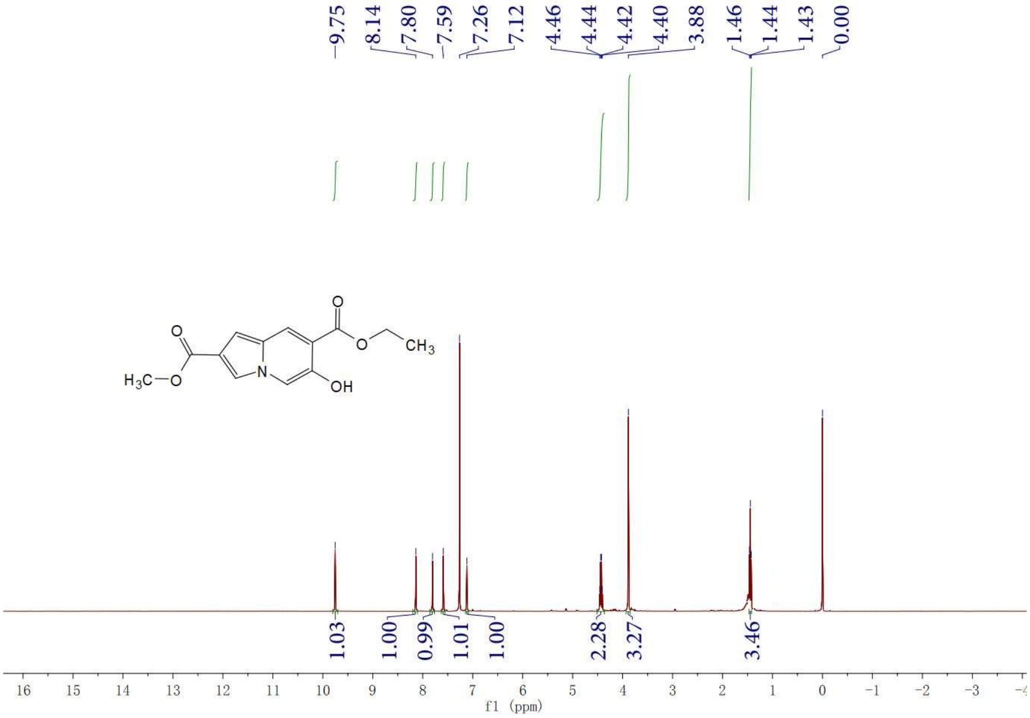
9.75
8.14
7.80
7.59
7.26
7.12
4.46
4.44
4.42
4.40
3.88
1.46
1.44
1.43
0.00
O
O
CH3
H3C
N
OH
1.03
1.00
0.99
1.01
1.00
2.28
3.27
3.46
f1 (ppm)

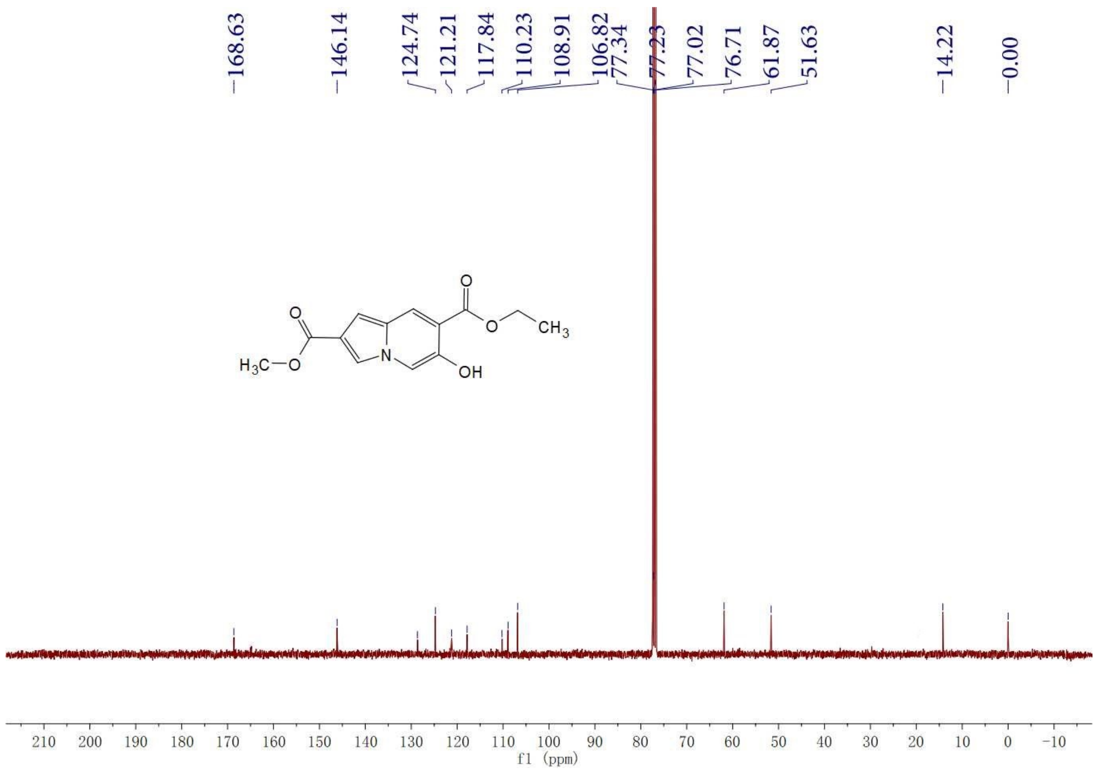

168.63
146.14
124.74
121.21
117.84
110.23
108.91
106.82
77.34
77.23
77.02
76.71
61.87
51.63
14.22
0.00
OH
CH3
H3C
N
f1 (ppm)


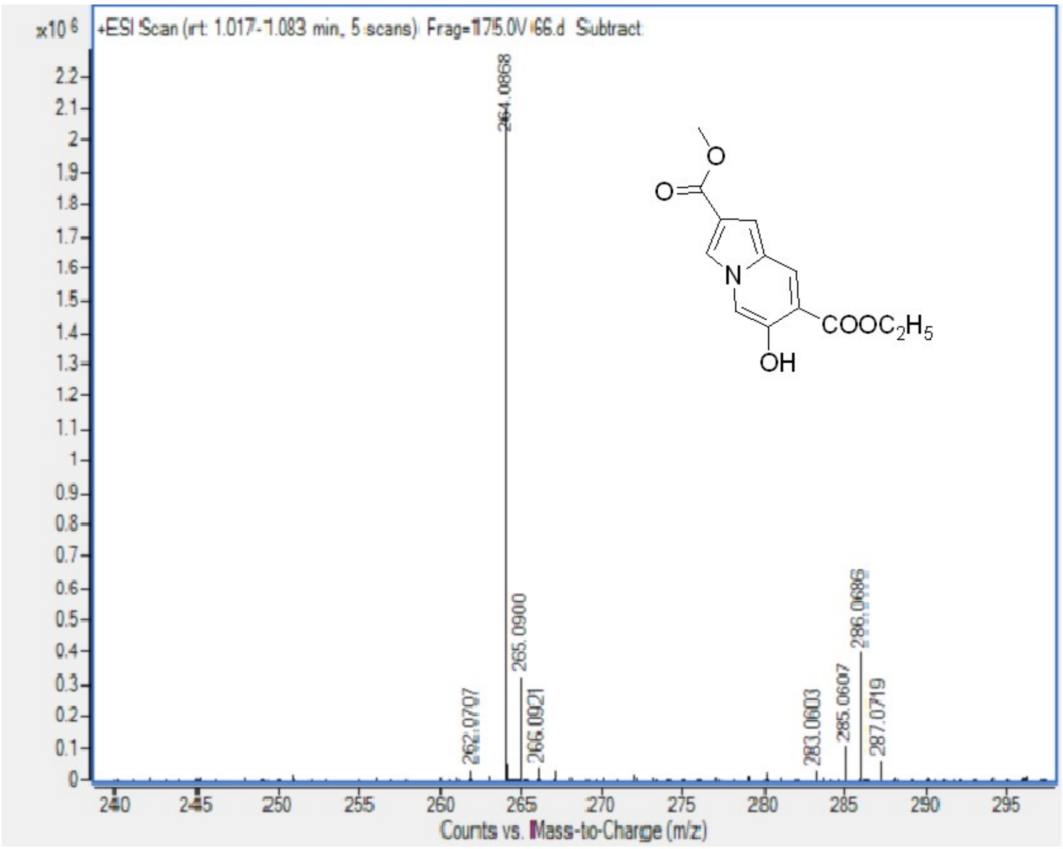

+ESI Scan (rt: 1.017-1.083 min, 5 scans) Frag=175.0V 66.d Subtract
x10 6
264.0868
265.0900
266.0921
262.0707
286.0686
285.0607
287.0719
283.0603
COOC2H5
OH
N
Counts vs. Mass-to-Charge (m/z)

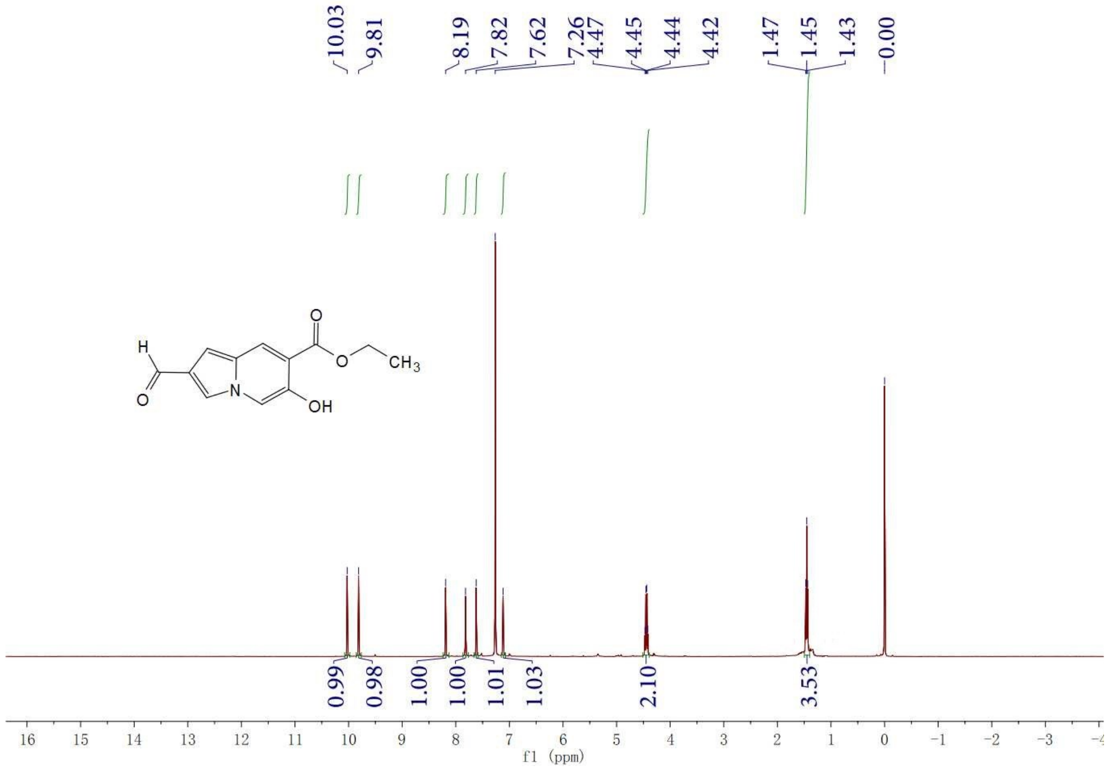
10.03
9.81
8.19
7.82
7.62
7.26
4.47
4.45
4.44
4.42
1.47
1.45
1.43
0.00
H
O
O
O
CH3
N
OH
0.99
0.98
1.00
1.00
1.01
1.03
2.10
3.53
f1 (ppm)

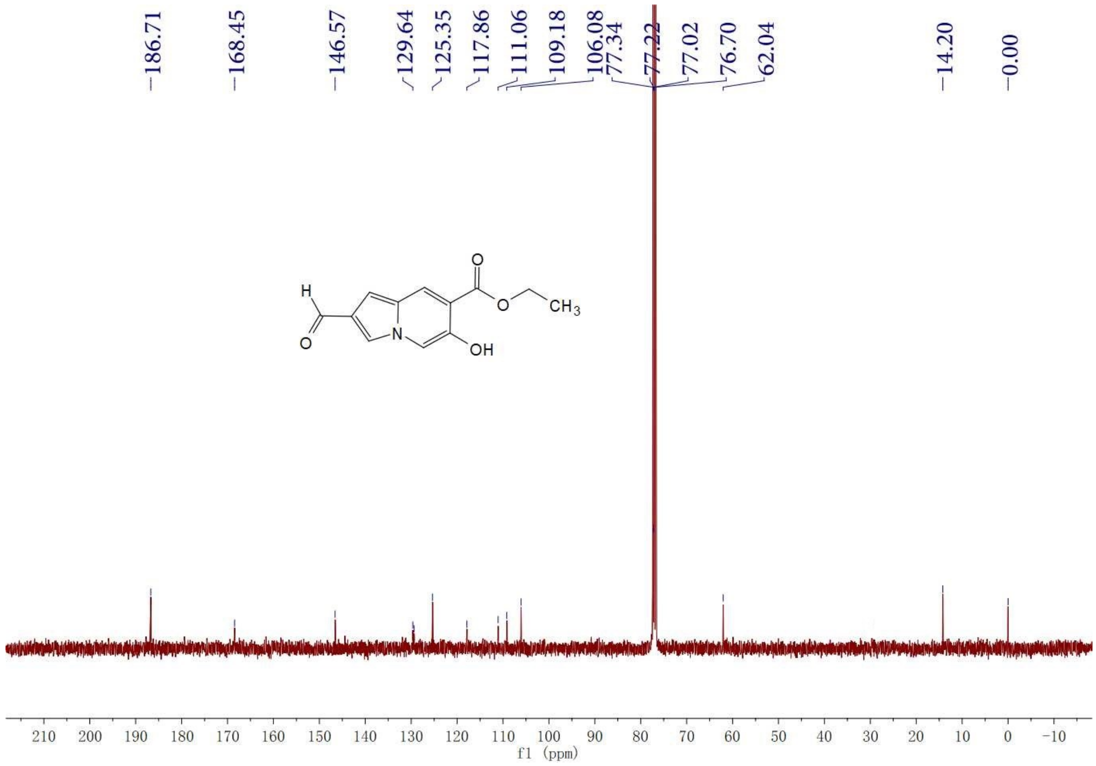
186.71
168.45
146.57
129.64
125.35
117.86
111.06
109.18
106.08
77.34
77.22
77.02
76.70
62.04
14.20
0.00
H
O
O
O
CH3
N
OH
f1 (ppm)

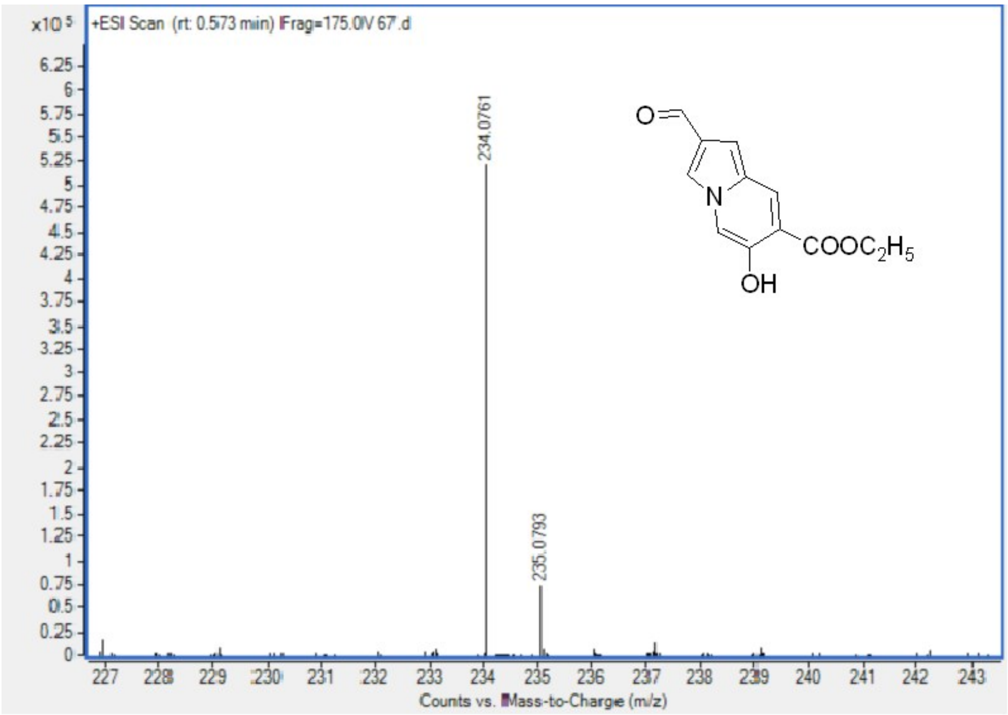
+ESI Scan (rt: 0.573 min) Frag=175.0V 67.d
x10 5
234.0761
235.0793
COOC2H5
OH
N
O
Counts vs. Mass-to-Charge (m/z)

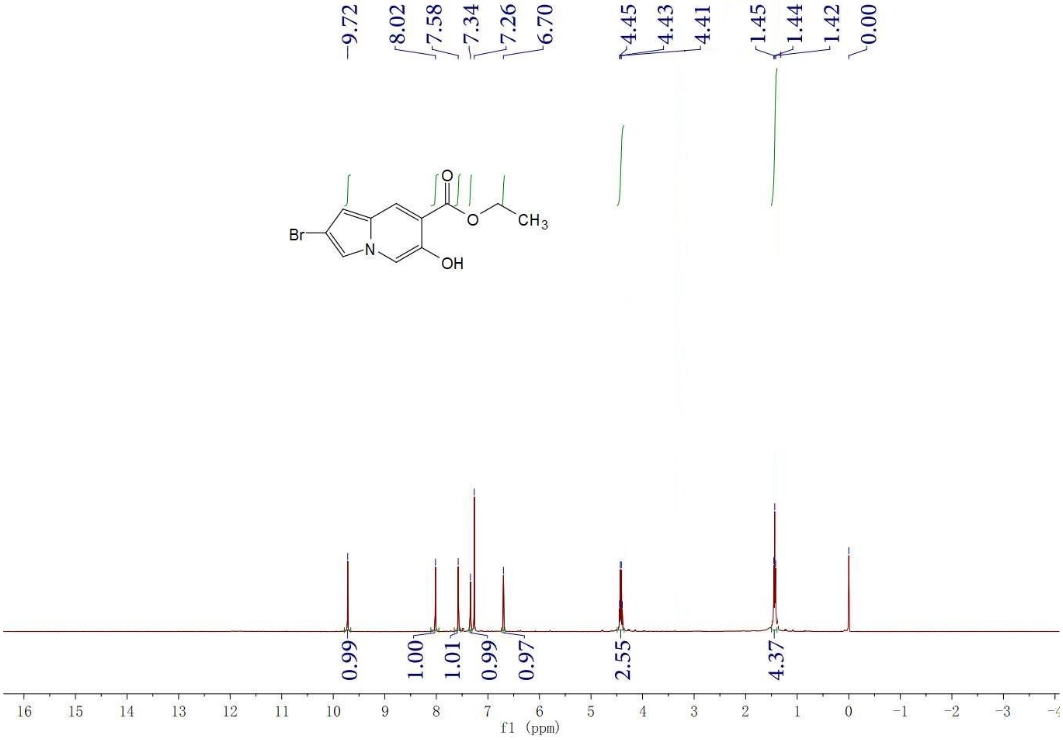
9.72
8.02
7.58
7.34
7.26
6.70
4.45
4.43
4.41
1.45
1.44
1.42
0.00
Br
N
OH
O
CH3
0.99
1.00
1.01
0.99
0.97
2.55
4.37
f1 (ppm)

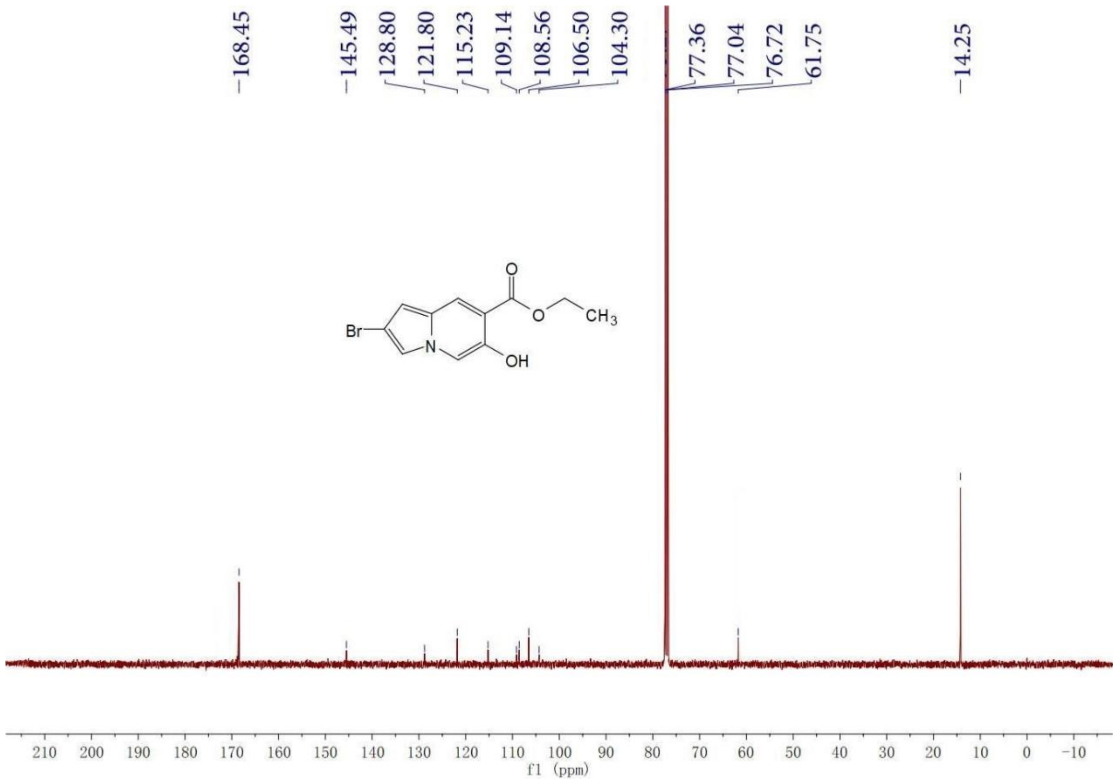

168.45
145.49
128.80
121.80
115.23
109.14
108.56
106.50
104.30
77.36
77.04
76.72
61.75
14.25
Br
N
O
O
CH3
OH
f1 (ppm)


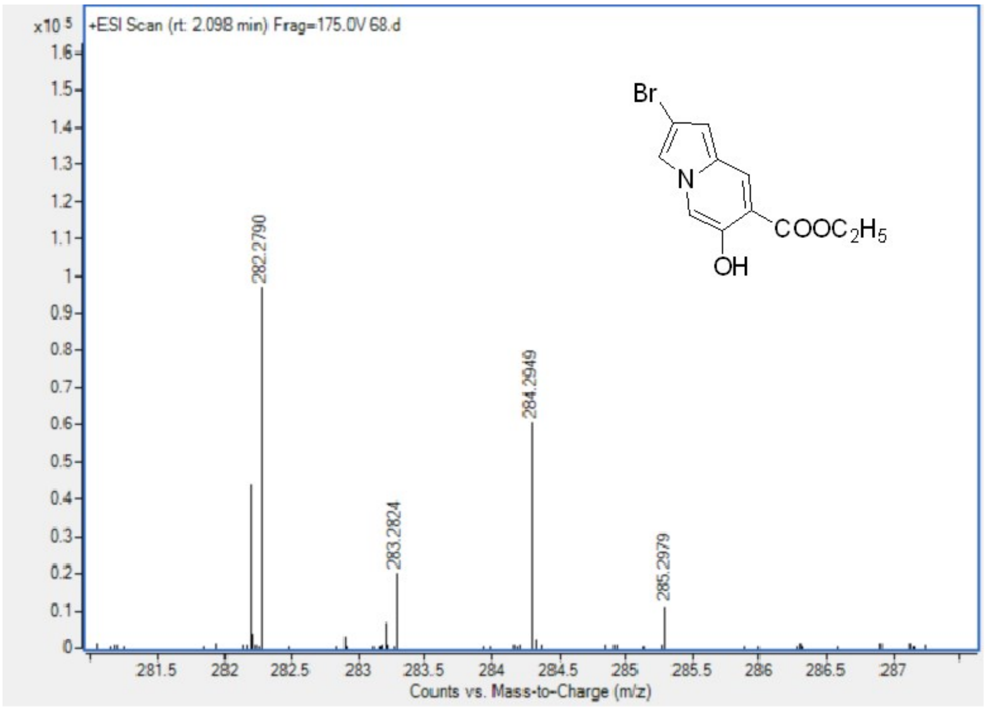

x10 5
+ESI Scan (rt: 2.098 min) Frag=175.0V 68.d
282.2790
283.2824
284.2949
285.2979
Br
N
COOC2H5
OH
Counts vs. Mass-to-Charge (m/z)

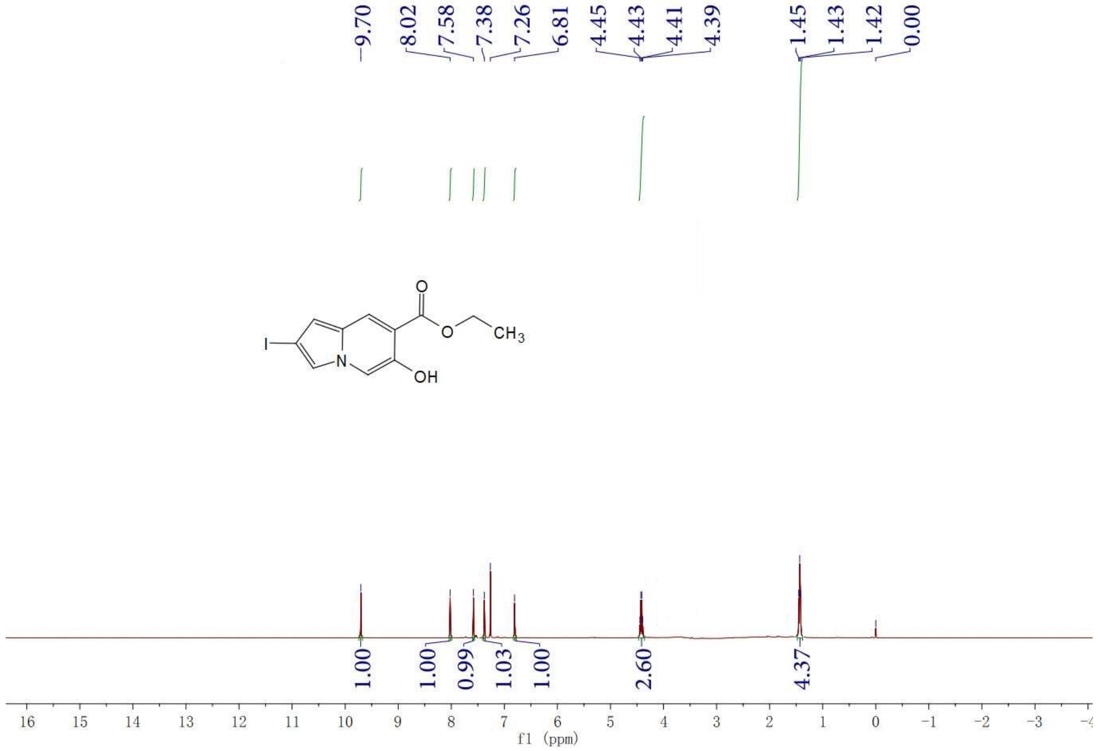
9.70 8.02 7.58 7.38 7.26 6.81 4.45 4.43 4.41 4.39 1.45 1.43 1.42 0.00
1.00 1.00 0.99 1.03 1.00 2.60 4.37
OH
CH3
f1 (ppm)

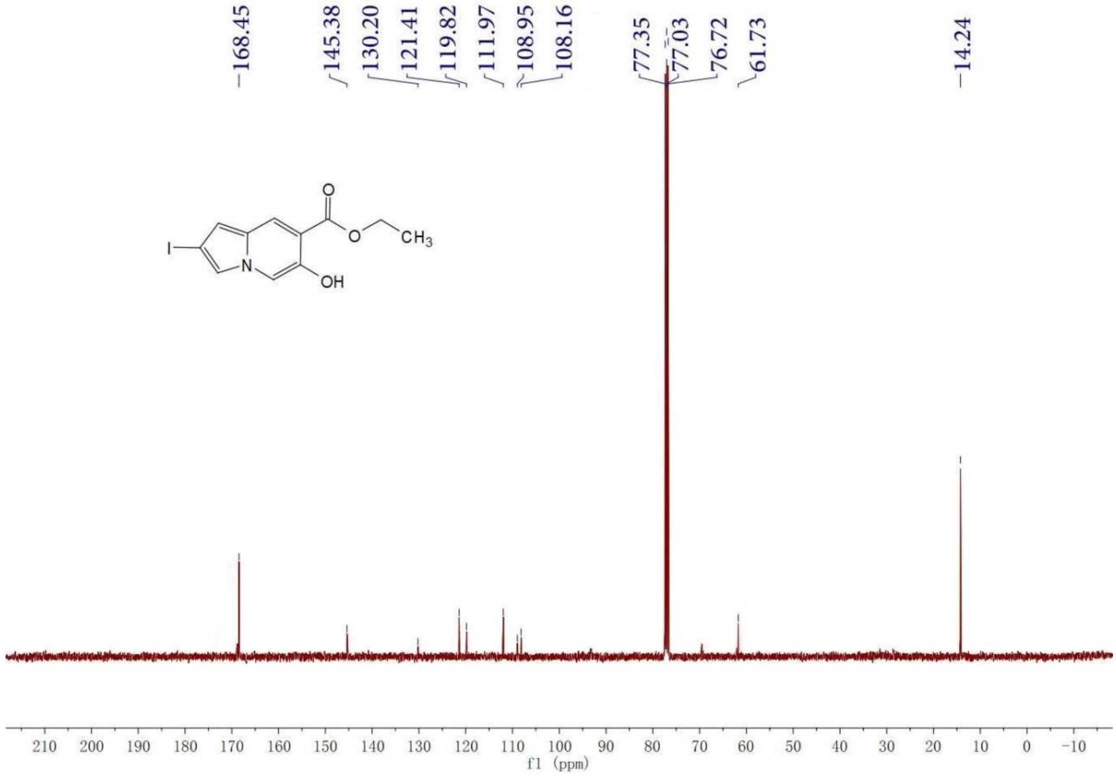
168.45 145.38 130.20 121.41 119.82 111.97 108.95 108.16 77.35 77.03 76.72 61.73 14.24
OH
CH3
f1 (ppm)

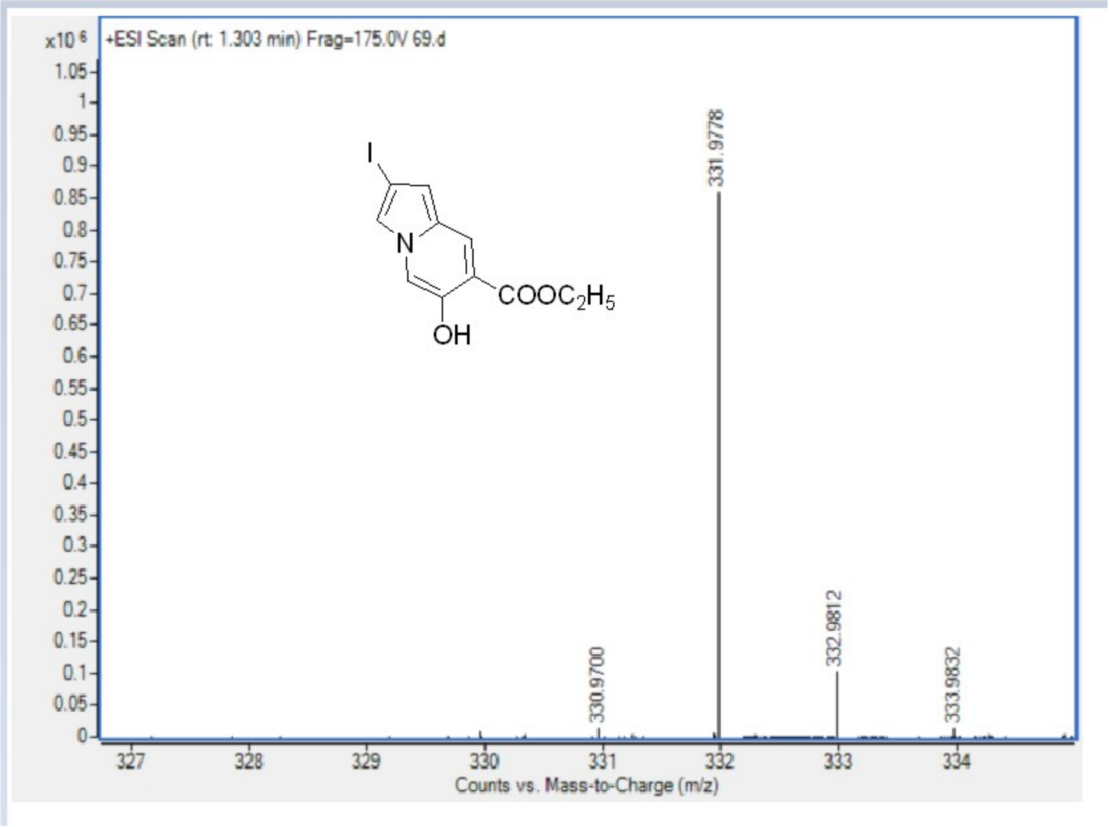
x10 6
+ESI Scan (rt: 1.303 min) Frag=175.0V 69.d
331.9778
332.9812
330.9700
333.9832
COOC2H5
OH
I
N
Counts vs. Mass-to-Charge (m/z)

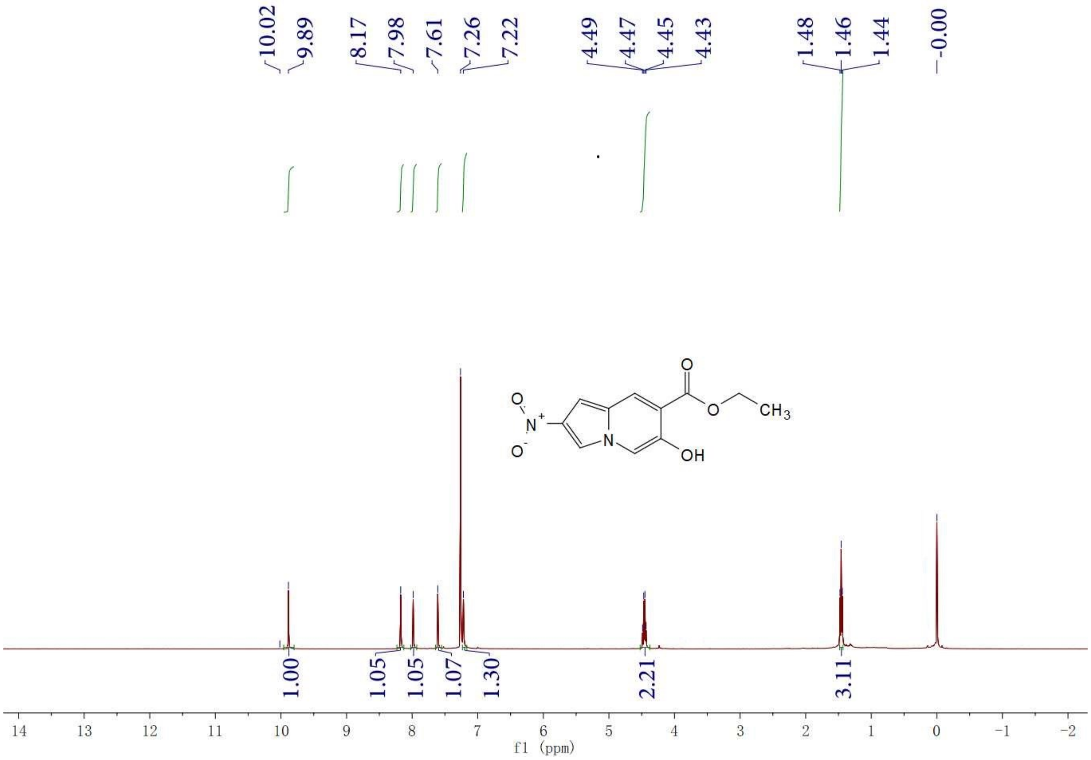
10.02
9.89
8.17
7.98
7.61
7.26
7.22
4.49
4.47
4.45
4.43
1.48
1.46
1.44
-0.00
1.00
1.05
1.05
1.07
1.30
2.21
3.11
OH
CH3
f1 (ppm)

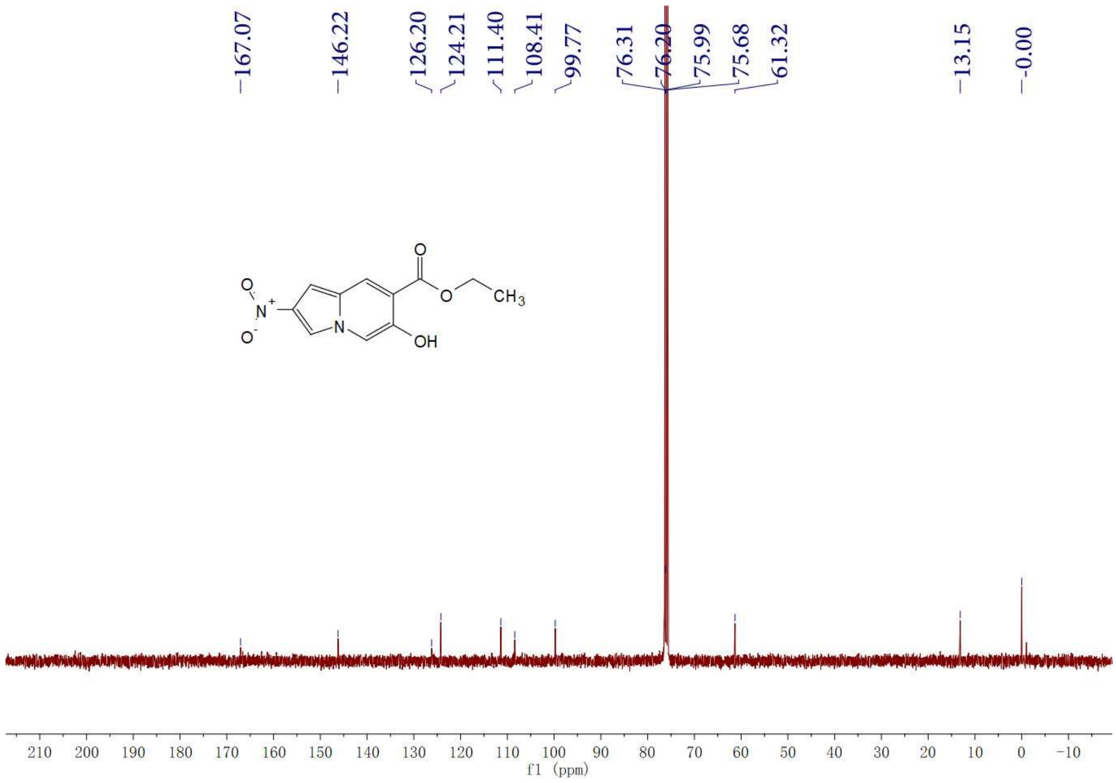

167.07
146.22
126.20
124.21
111.40
108.41
99.77
76.31
76.20
75.99
75.68
61.32
13.15
-0.00
CH3
OH
f1 (ppm)


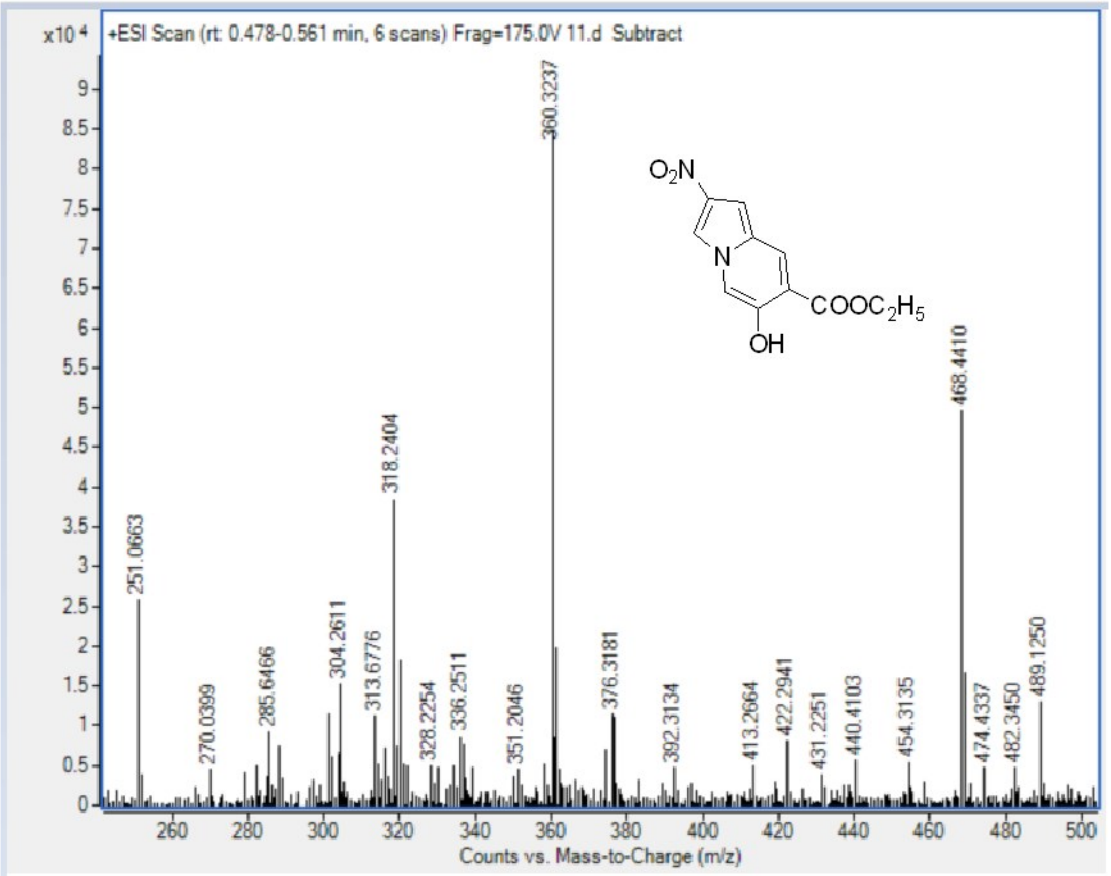

+ESI Scan (rt: 0.478-0.561 min, 6 scans) Frag=175.0V 11.d Subtract
x10 4
251.0663
270.0399
285.6466
304.2611
313.6776
318.2404
328.2254
336.2511
351.2046
360.3237
376.3181
392.3134
413.2664
422.2941
431.2251
440.4103
454.3135
468.4410
474.4337
482.3450
489.1250
O2N
COOC2H5
OH
Counts vs. Mass-to-Charge (m/z)

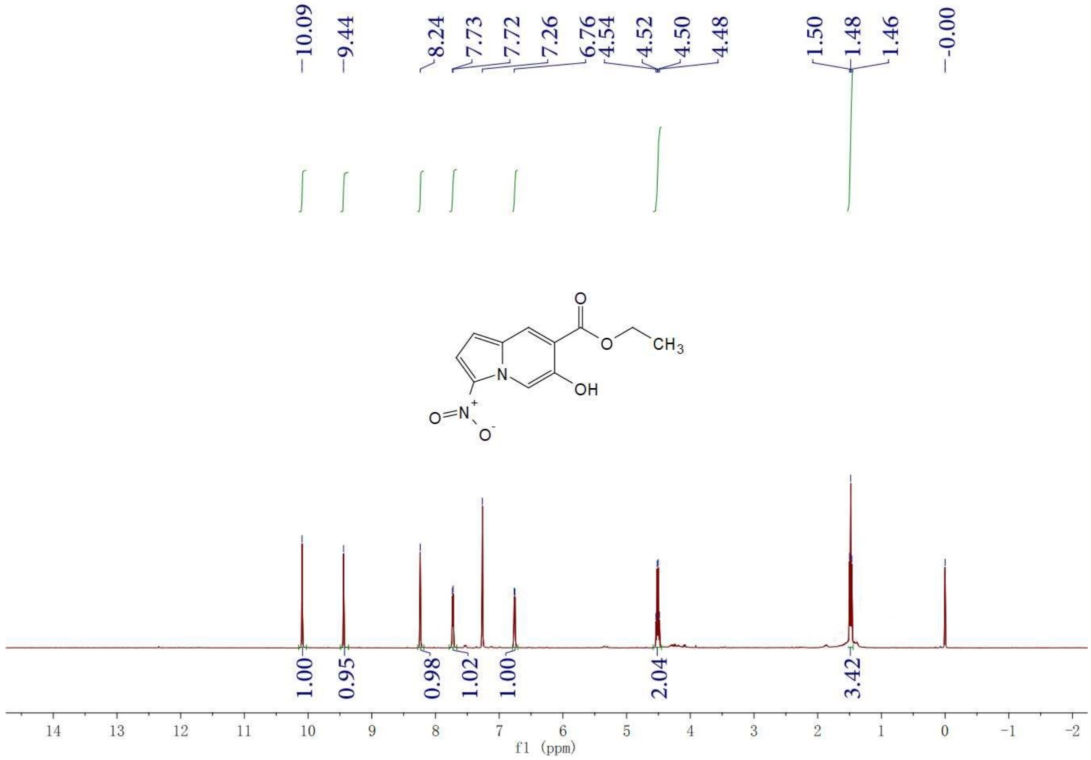
10.09
9.44
8.24
7.73
7.72
7.26
6.76
4.54
4.52
4.50
4.48
1.50
1.48
1.46
-0.00
$CH_3$
OH
N
O
1.00
0.95
0.98
1.02
1.00
2.04
3.42
f1 (ppm)

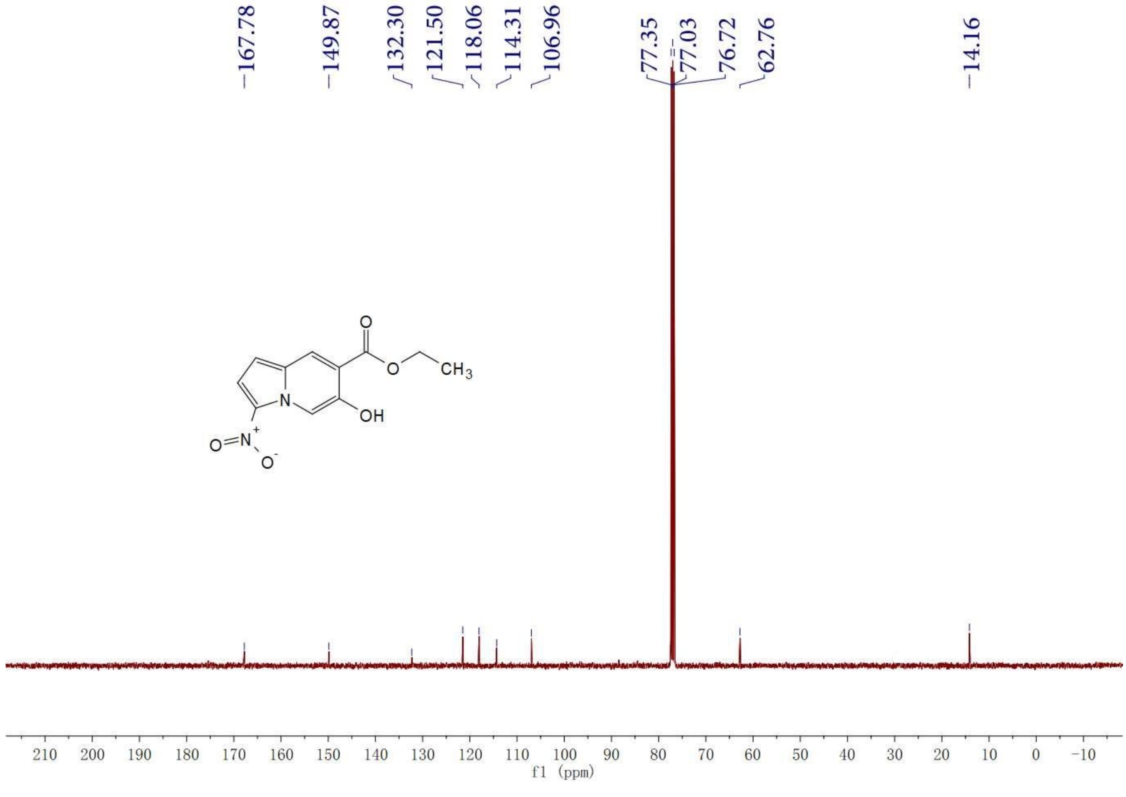
167.78
149.87
132.30
121.50
118.06
114.31
106.96
77.35
77.03
76.72
62.76
14.16
$CH_3$
OH
N
O
f1 (ppm)

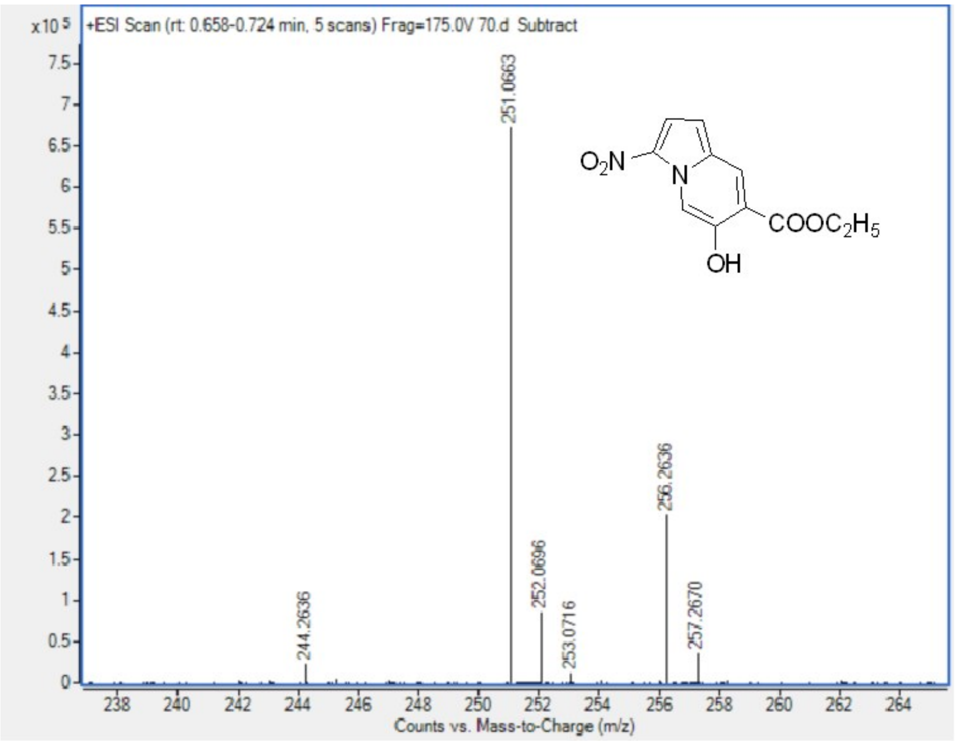
x10 5
+ESI Scan (rt: 0.658-0.724 min, 5 scans) Frag=175.0V 70.d Subtract
251.0663
252.0696
253.0716
244.2636
256.2636
257.2670
O2N
N
COOC2H5
OH
Counts vs. Mass-to-Charge (m/z)

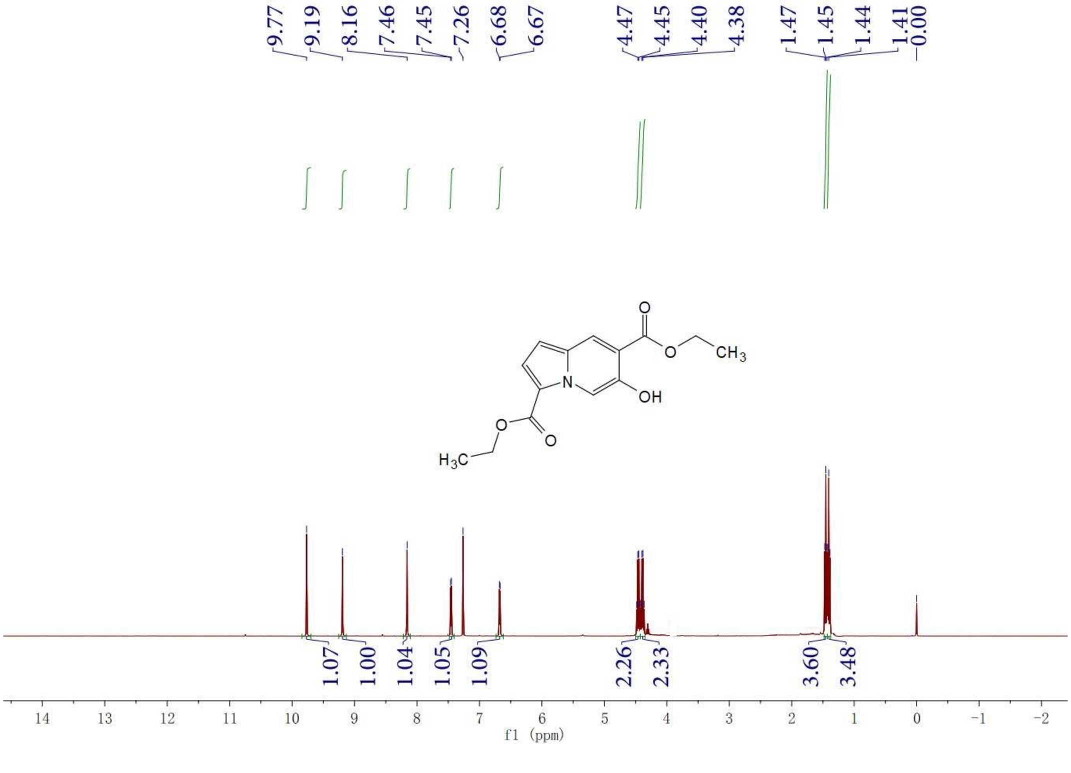
9.77
9.19
8.16
7.46
7.45
7.26
6.68
6.67
4.47
4.45
4.40
4.38
1.47
1.45
1.44
1.41
0.00
O
CH3
N
OH
H3C
O
1.07
1.00
1.04
1.05
1.09
2.26
2.33
3.60
3.48
f1 (ppm)

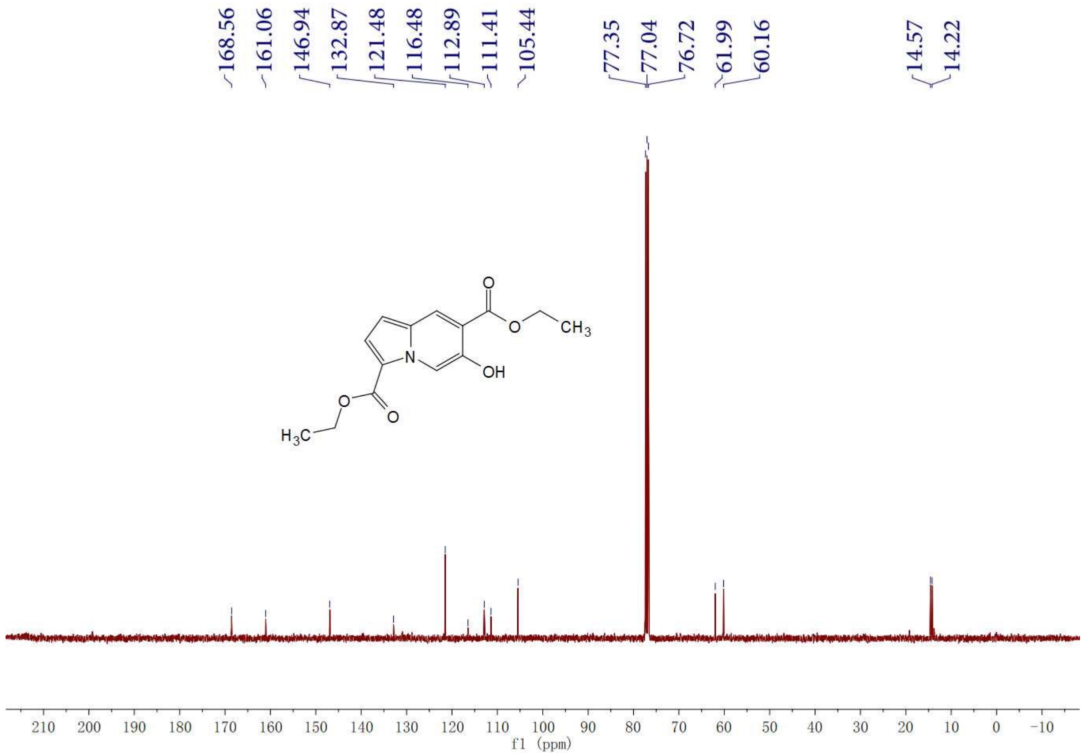

168.56
161.06
146.94
132.87
121.48
116.48
112.89
111.41
105.44
77.35
77.04
76.72
61.99
60.16
14.57
14.22
O
CH3
N
OH
O
O
H3C
210 200 190 180 170 160 150 140 130 120 110 100 90 80 70 60 50 40 30 20 10 0 −10
f1 (ppm)


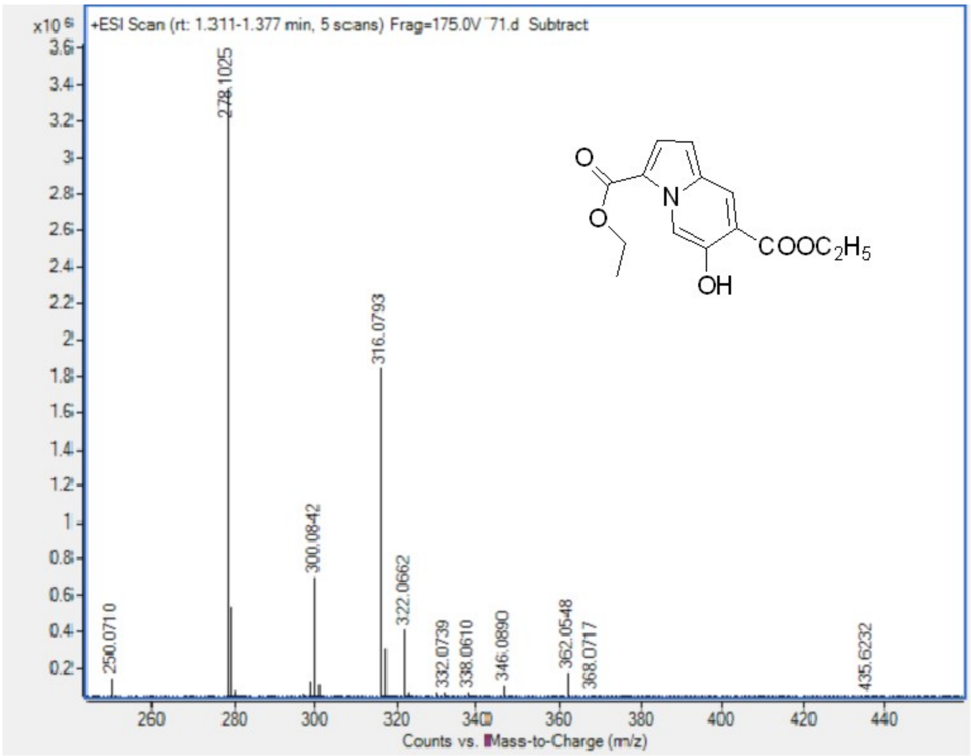

+ESI Scan (rt: 1.311-1.377 min, 5 scans) Frag=175.0V 71.d Subtract
278.1025
316.0793
300.0842
322.0662
250.0710
332.0739
338.0610
346.0890
362.0548
368.0717
435.6232
COOC2H5
OH
N
O
O
Counts vs. Mass-to-Charge (m/z)

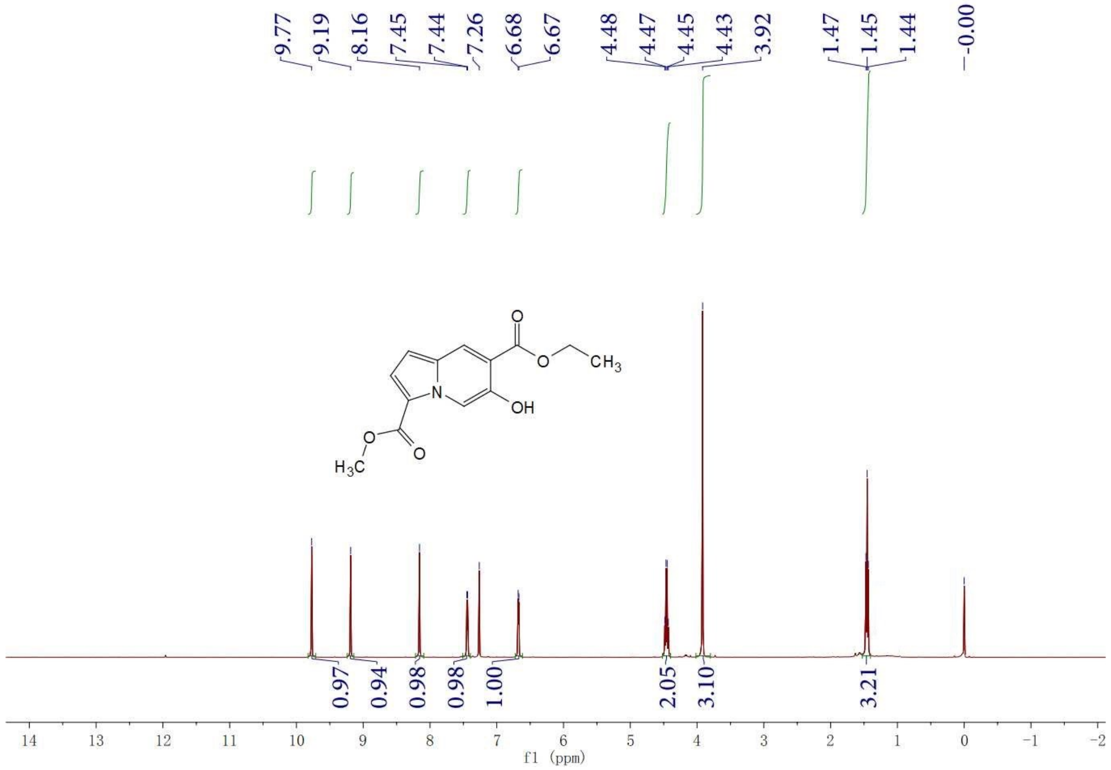
9.77
9.19
8.16
7.45
7.44
7.26
6.68
6.67
4.48
4.47
4.45
4.43
3.92
1.47
1.45
1.44
-0.00
OH
CH3
H3C
N
O
0.97
0.94
0.98
0.98
1.00
2.05
3.10
3.21
f1 (ppm)

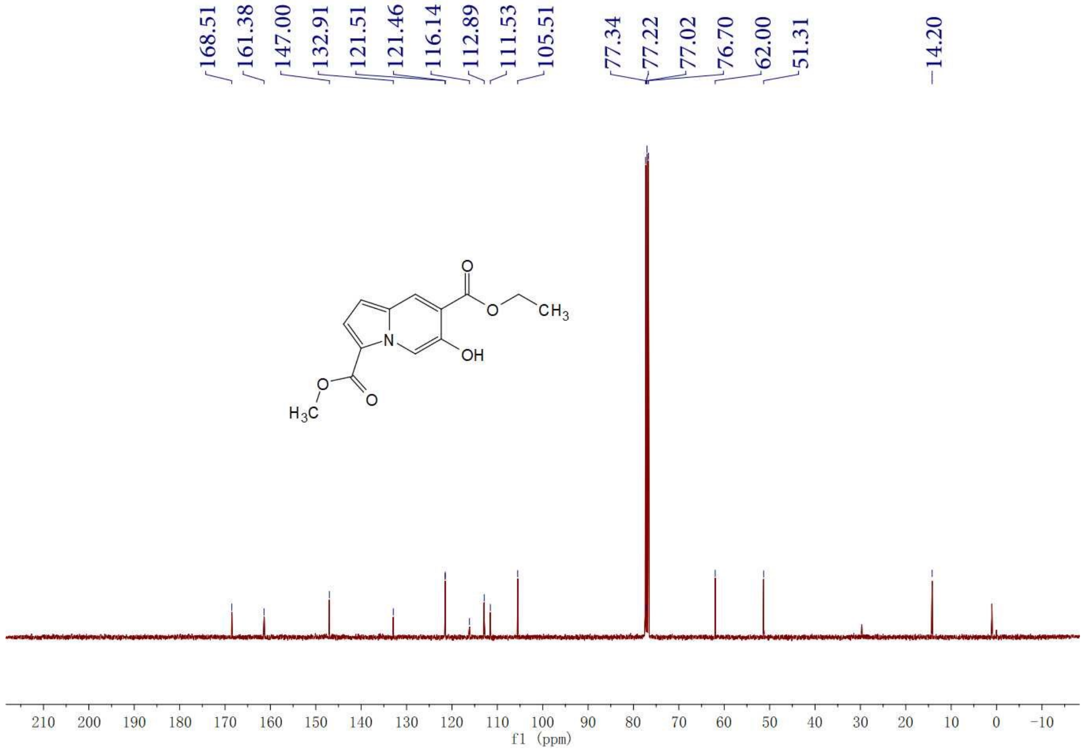
168.51
161.38
147.00
132.91
121.51
121.46
116.14
112.89
111.53
105.51
77.34
77.22
77.02
76.70
62.00
51.31
14.20
OH
CH3
H3C
N
O
f1 (ppm)

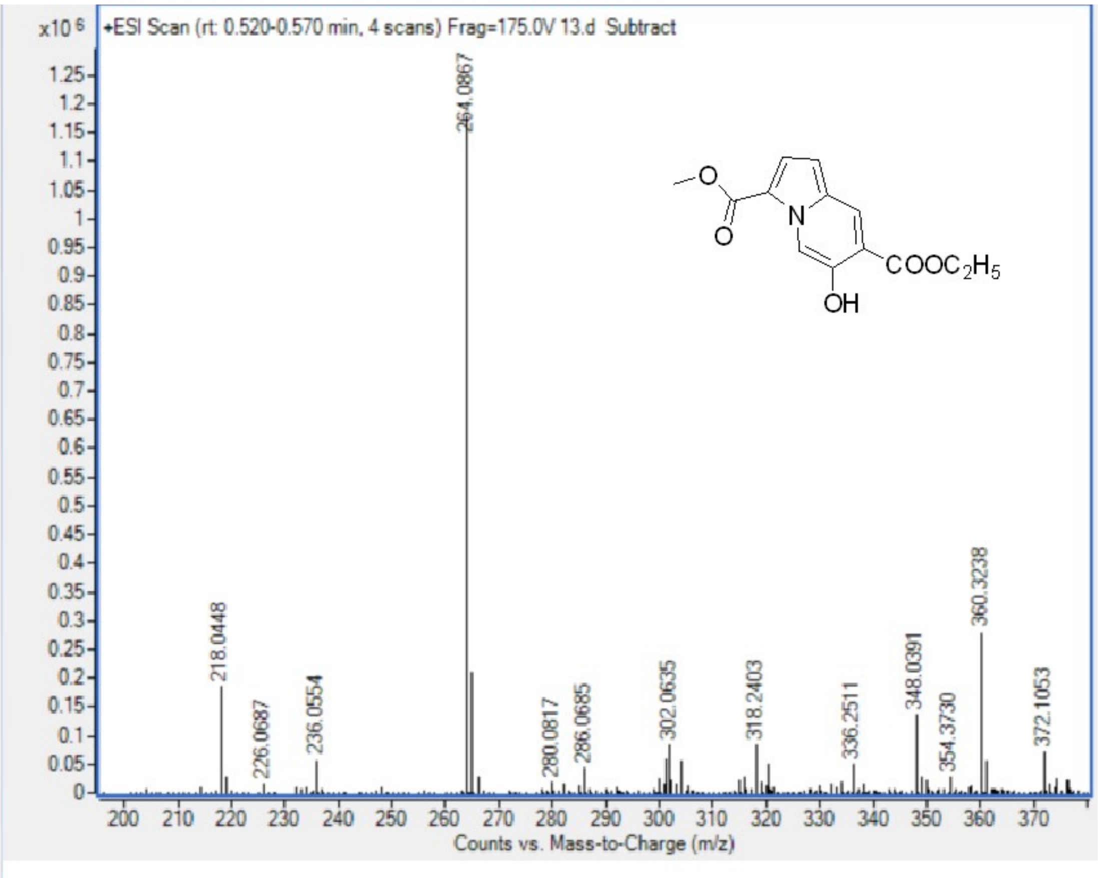

x10 6
+ESI Scan (rt: 0.520-0.570 min, 4 scans) Frag=175.0V 13.d Subtract
264.0867
218.0448
226.0687
236.0554
280.0817
286.0685
302.0635
318.2403
336.2511
348.0391
354.3730
360.3238
372.1053
COOC2H5
OH
N
O
Counts vs. Mass-to-Charge (m/z)


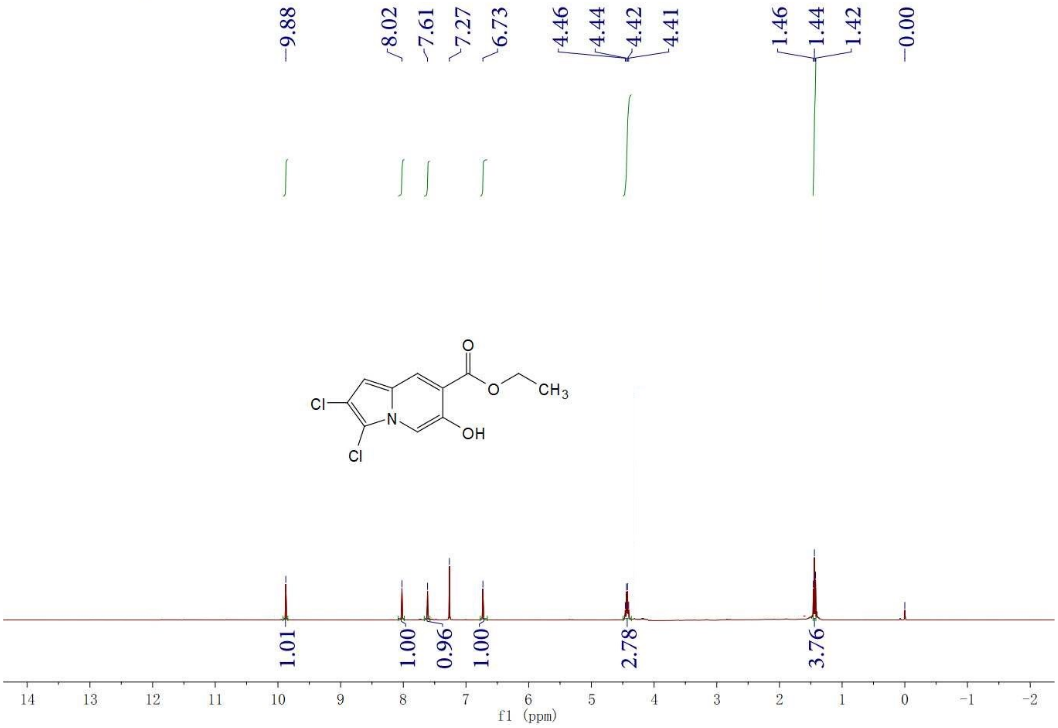

9.88
8.02
7.61
7.27
6.73
4.46
4.44
4.42
4.41
1.46
1.44
1.42
0.00
Cl
Cl
N
O
CH3
OH
1.01
1.00
0.96
1.00
2.78
3.76
f1 (ppm)

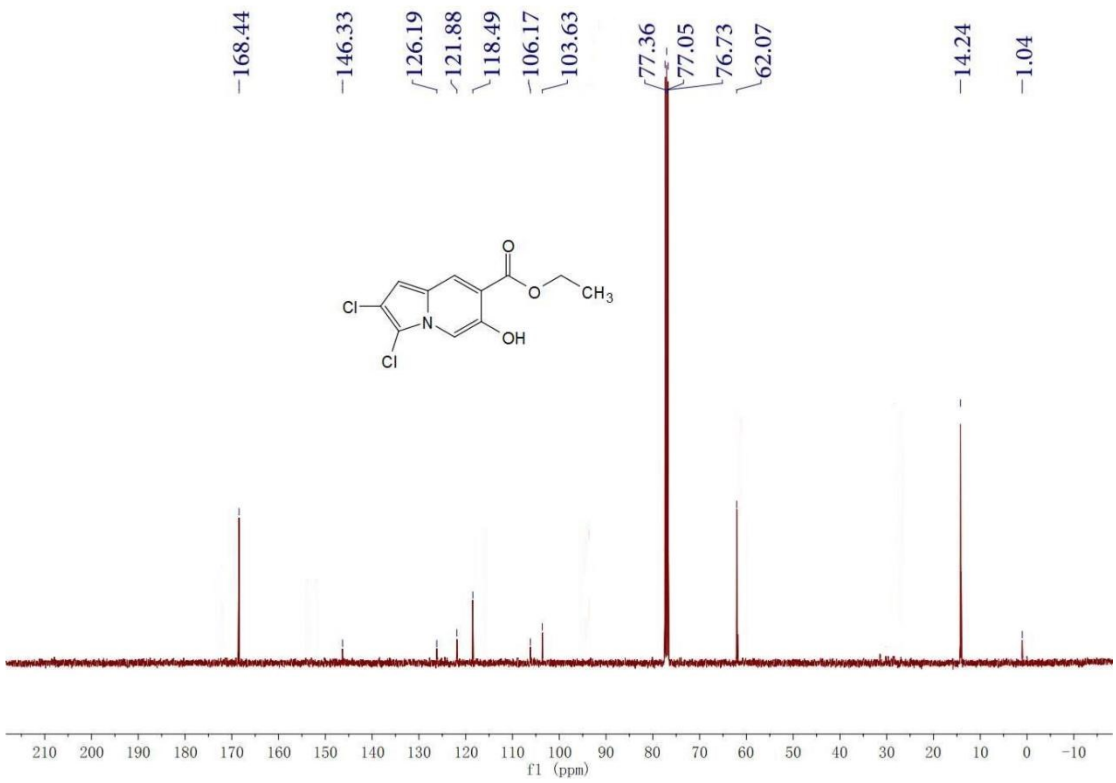

168.44
146.33
126.19
121.88
118.49
106.17
103.63
77.36
77.05
76.73
62.07
14.24
1.04
Cl
Cl
N
O
O
CH3
OH
f1 (ppm)


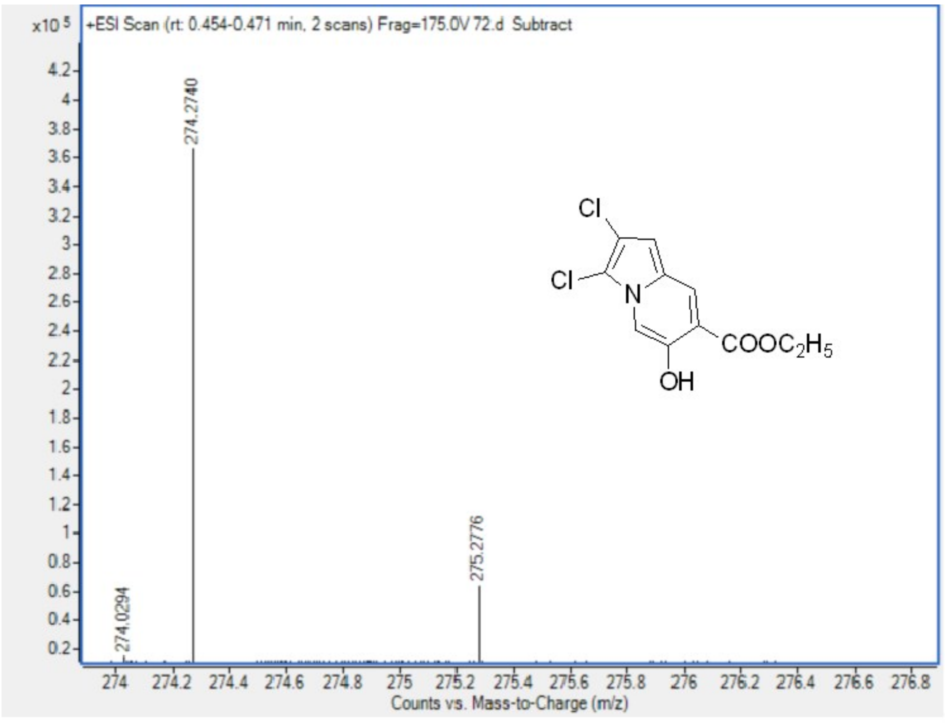

x10 5
+ESI Scan (rt: 0.454-0.471 min, 2 scans) Frag=175.0V 72.d Subtract
274.2740
275.2776
274.0294
Cl
Cl
N
COOC2H5
OH
Counts vs. Mass-to-Charge (m/z)

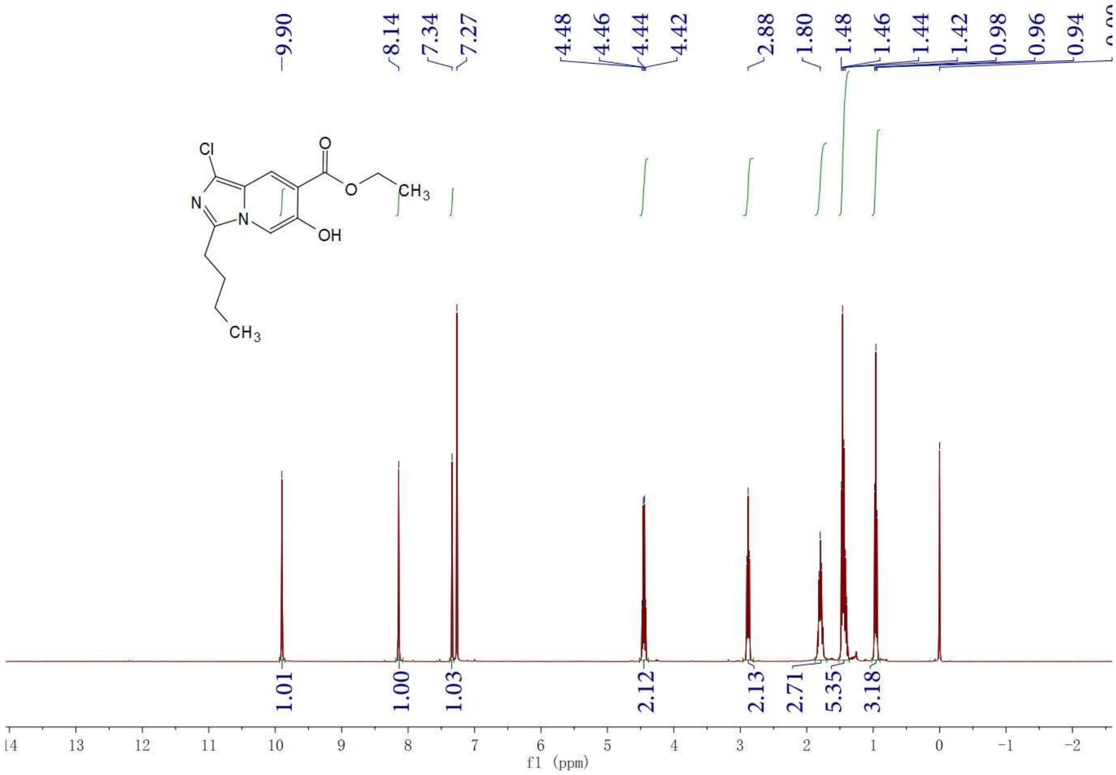
9.90
8.14
7.34
7.27
4.48
4.46
4.44
4.42
2.88
1.80
1.48
1.46
1.44
1.42
0.98
0.96
0.94
Cl
O
O
CH3
N
N
OH
CH3
1.01
1.00
1.03
2.12
2.13
2.71
5.35
3.18
f1 (ppm)

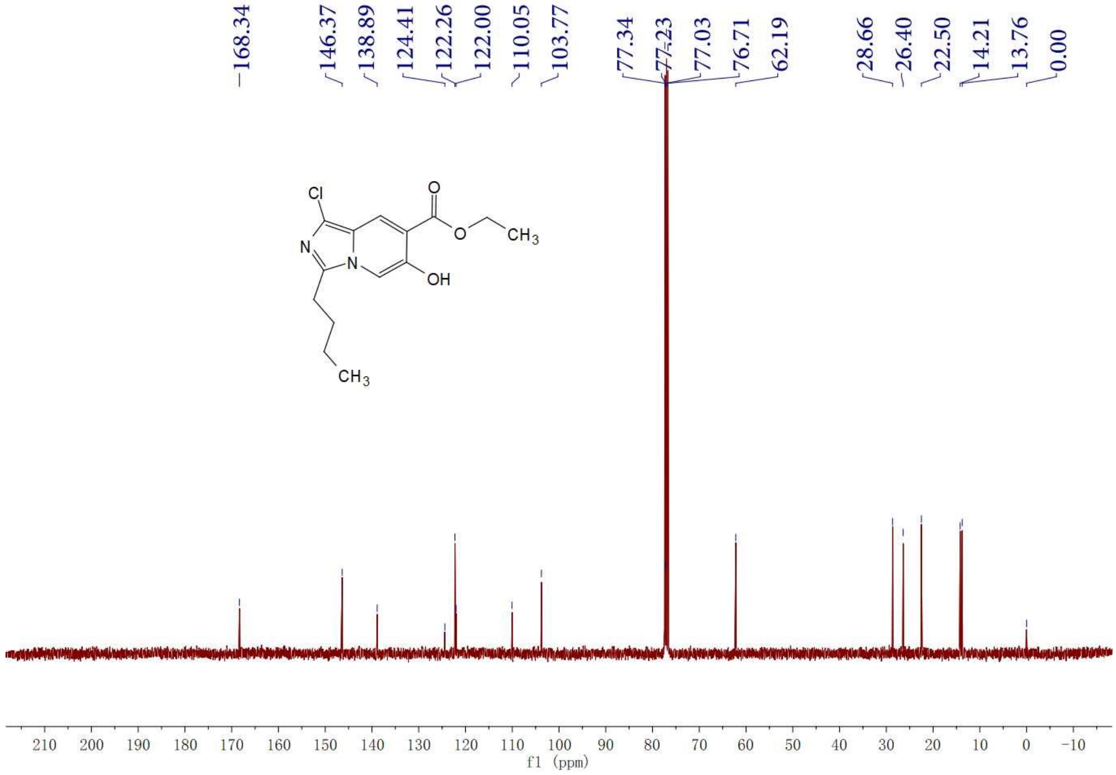
168.34
146.37
138.89
124.41
122.26
122.00
110.05
103.77
77.34
77.23
77.03
76.71
62.19
28.66
26.40
22.50
14.21
13.76
0.00
Cl
O
O
CH3
N
N
OH
CH3
f1 (ppm)

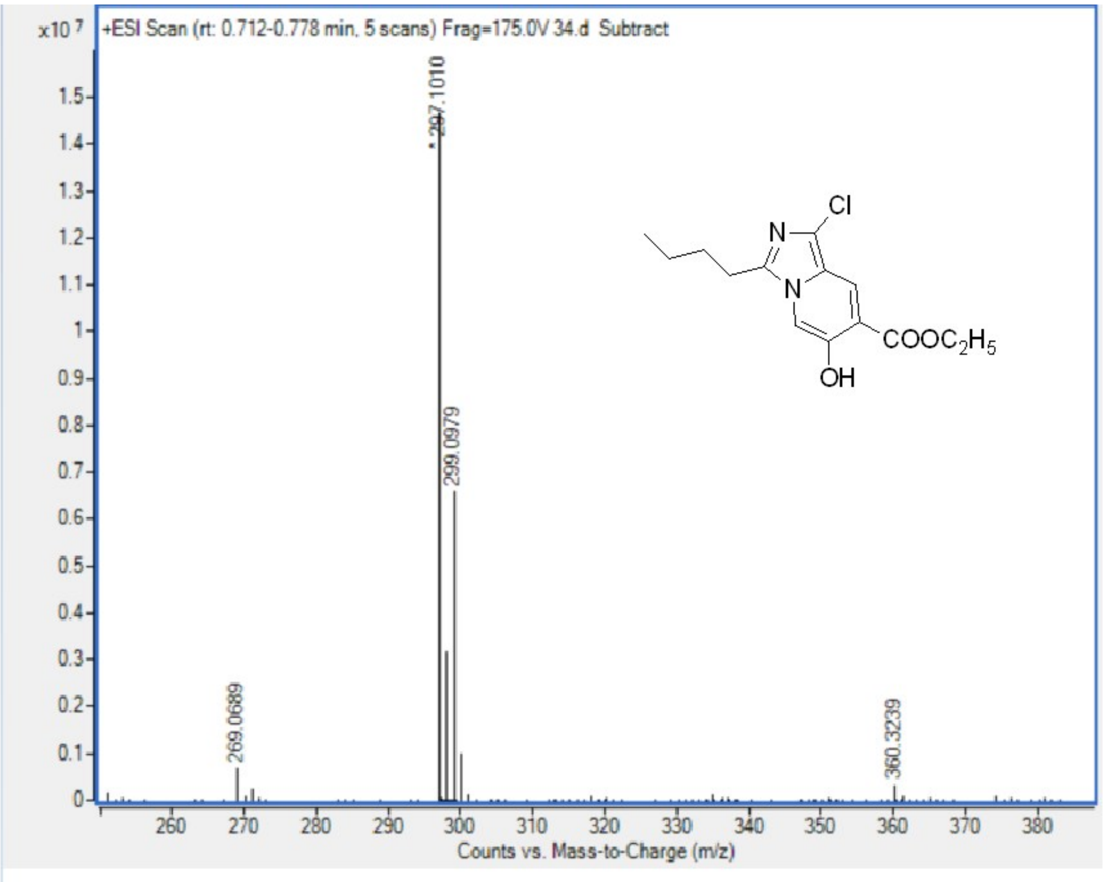
x10 7
+ESI Scan (rt: 0.712-0.778 min, 5 scans) Frag=175.0V 34.d Subtract
297.1010
299.0979
269.0689
360.3239
Cl
N
N
COOC2H5
OH
Counts vs. Mass-to-Charge (m/z)

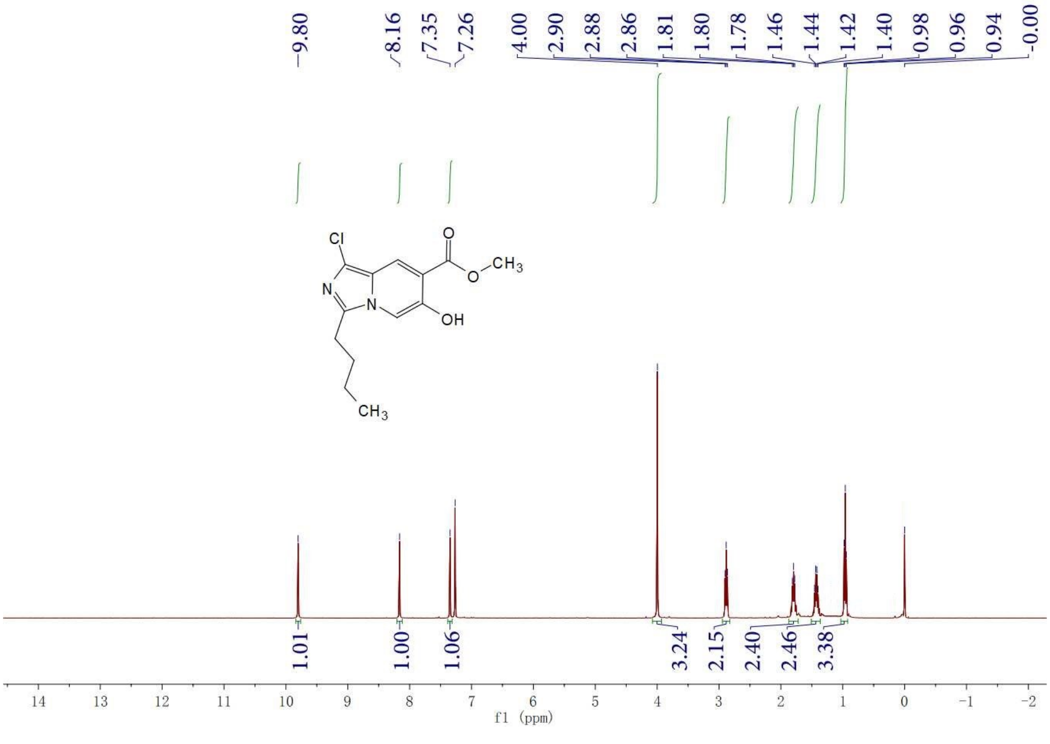
9.80
8.16
7.35
7.26
4.00
2.90
2.88
2.86
1.81
1.80
1.78
1.46
1.44
1.42
1.40
0.98
0.96
0.94
-0.00
Cl
O
CH3
N
N
OH
CH3
1.01
1.00
1.06
3.24
2.15
2.40
2.46
3.38
f1 (ppm)

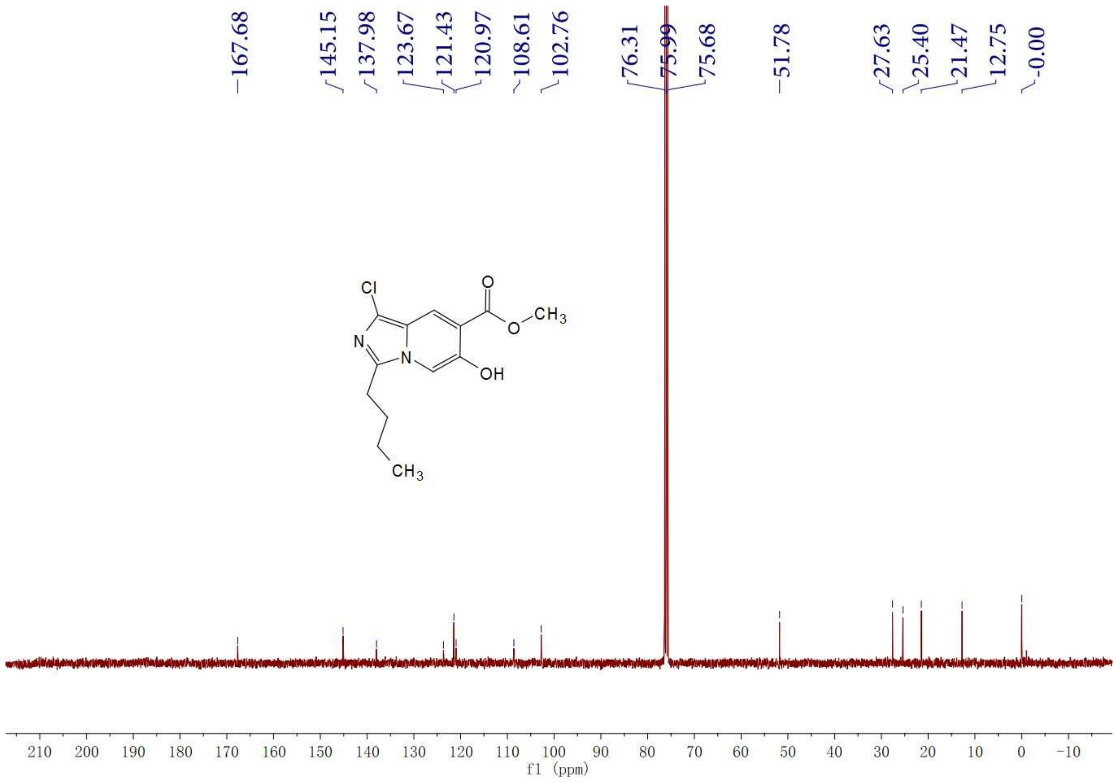

167.68
145.15
137.98
123.67
121.43
120.97
108.61
102.76
76.31
75.99
75.68
51.78
27.63
25.40
21.47
12.75
-0.00
Cl
O
N
N
O
CH3
OH
CH3
210 200 190 180 170 160 150 140 130 120 110 100 90 80 70 60 50 40 30 20 10 0 -10
f1 (ppm)


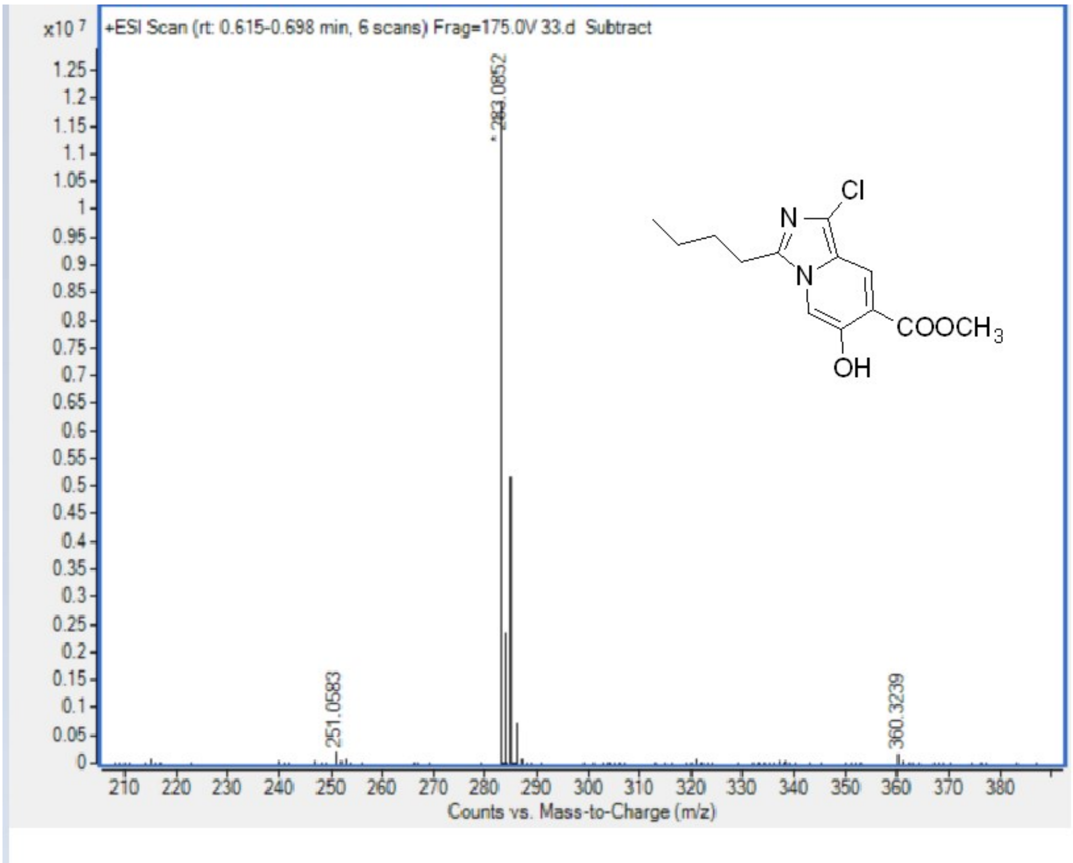

x10 7
+ESI Scan (rt: 0.615-0.698 min, 6 scans) Frag=175.0V 33.d Subtract
283.0852
251.0583
360.3239
Cl
N
N
COOCH3
OH
Counts vs. Mass-to-Charge (m/z)

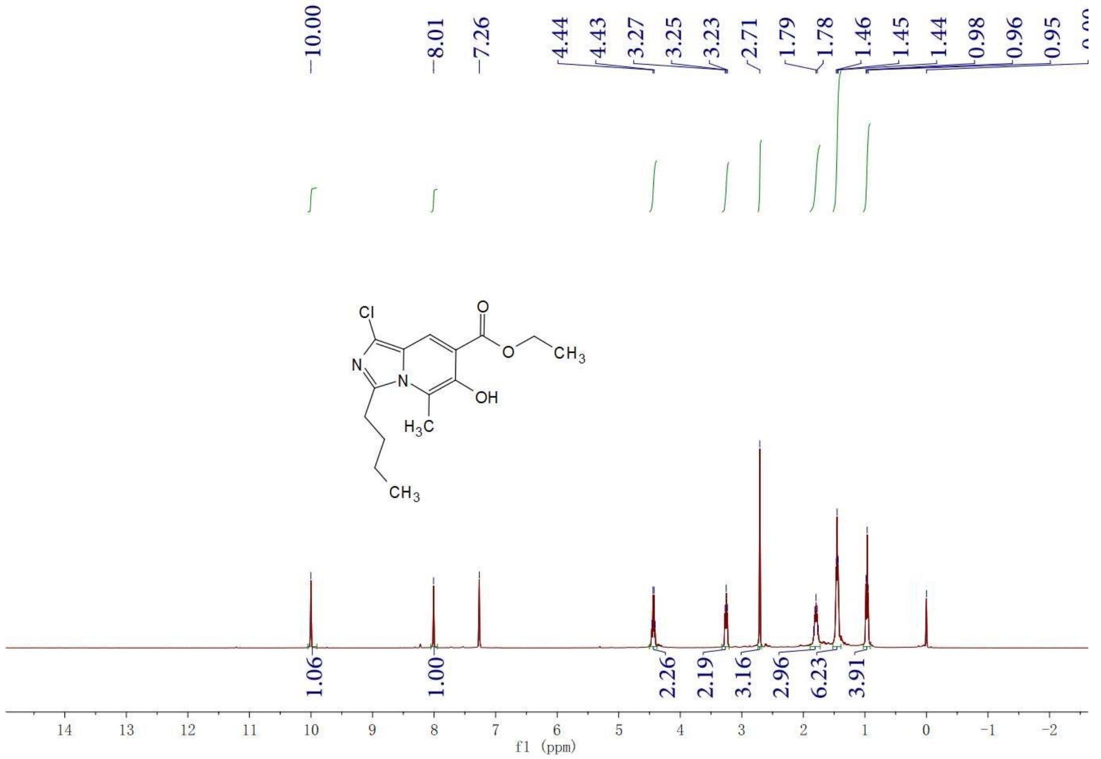

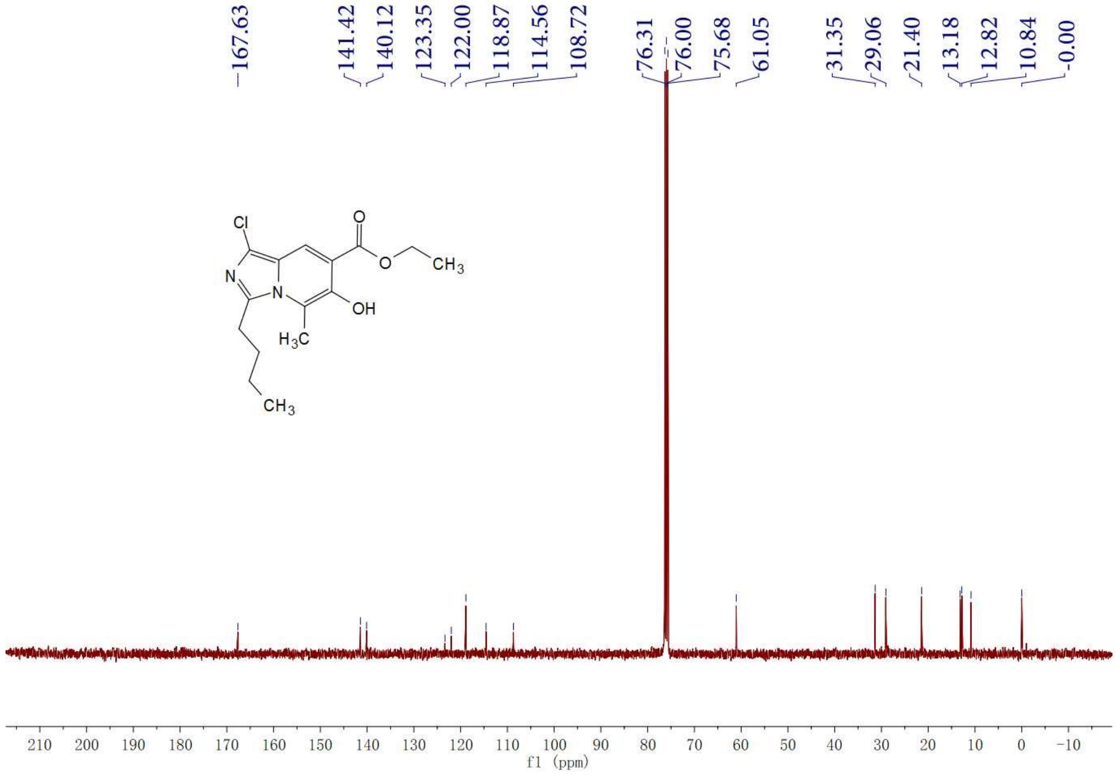

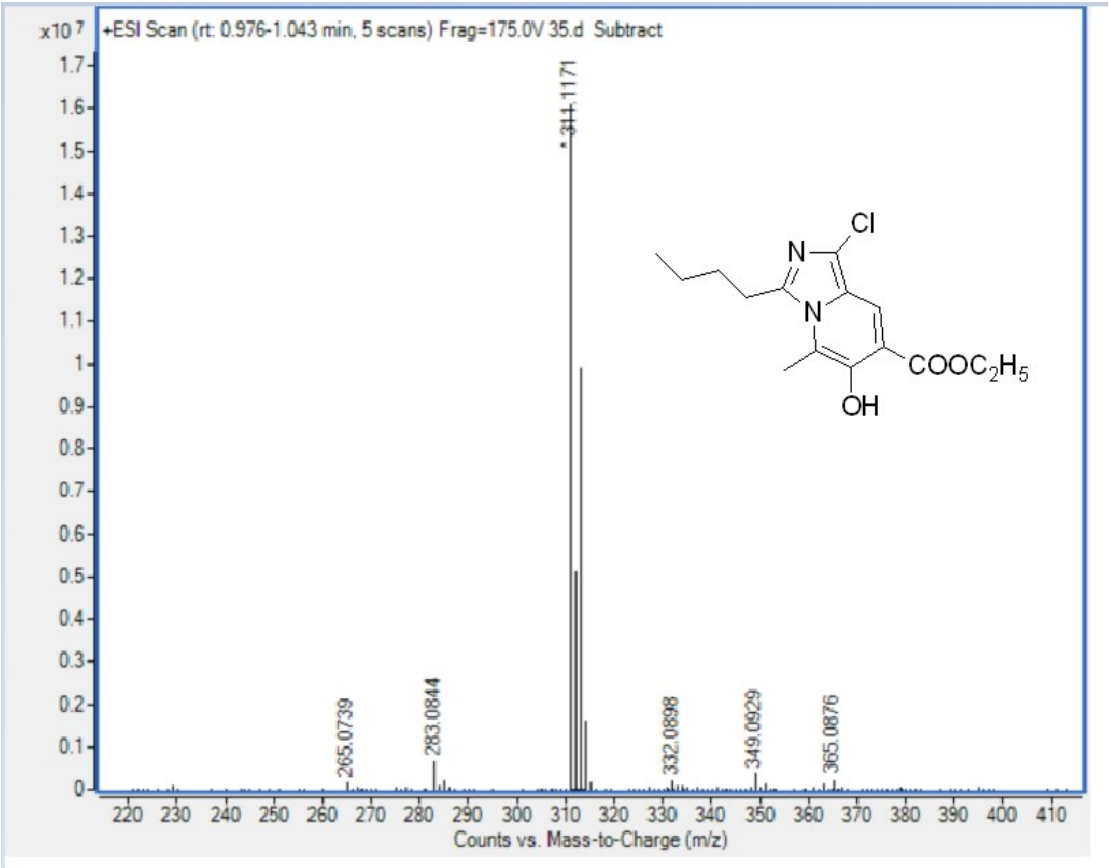

x10 7
+ESI Scan (rt: 0.976-1.043 min, 5 scans) Frag=175.0V 35.d Subtract
311.1171
265.0739
283.0844
332.0898
349.0929
365.0876
Counts vs. Mass-to-Charge (m/z)
Cl
N
N
COOC2H5
OH
